# Mapping functional connectivity in the pigeon brain with wide-field optical imaging

**DOI:** 10.1117/1.NPh.13.1.015010

**Published:** 2026-02-06

**Authors:** Kathryn C. Chenard, Annie R. Bice, Seana H. Bice, Joseph P. Culver, Paula Gerliz, Xavier Helluy, Onur Güntürkün, Jason W. Trobaugh, Mehdi Behroozi, Carlos A. Botero

**Affiliations:** a University of Texas at Austin, Department of Integrative Biology, Austin, Texas, United States; b Washington University School of Medicine, Department of Radiology, St. Louis, Missouri, United States; c Washington University in St. Louis, Department of Biomedical Engineering, St. Louis, Missouri, United States; d Washington University in St. Louis, Department of Physics, St. Louis, Missouri, United States; e Washington University in St. Louis, Department of Neuroscience, St. Louis, Missouri, United States; f Ruhr University Bochum, Institute of Cognitive Neuroscience, Department of Biopsychology, Bochum, Germany; g Washington University in St. Louis, Department of Electrical and Systems Engineering, St. Louis, Missouri, United States

**Keywords:** optical intrinsic signal imaging, widefield optical imaging, pigeon, functional connectivity, brain, resting state

## Abstract

**Significance:**

Adapting optical imaging technology to avian models can overcome many limitations imposed by functional magnetic resonance imaging (fMRI), which currently restricts the number of species used to study functional connectivity. Developing advanced technology to expand the diversity of species that can be effectively imaged is crucial for addressing significant questions that are currently unreachable, such as understanding the evolution of cognition from a comparative perspective.

**Aim:**

We assessed the potential of optical imaging technology to measure functional connectivity in birds, utilizing pigeons as an avian model. We evaluated whether we could partition the dorsal surface of the pigeon brain into units that correspond to known anatomical regions. Finally, we compared our results with those obtained from a separate dataset acquired using fMRI.

**Approach:**

Using optical intrinsic signal imaging, a widefield optical imaging method, we imaged resting state functional connectivity in scalp-retracted anesthetized pigeons. We then used iterative parcellation and hierarchical clustering to create functional connectivity maps of correlation between parcels at two spatial scales. We recorded a second independent dataset of ten pigeons using a single-shot multi-slice gradient echo EPI sequence fMRI and applied the same parcellation method to compare functional connectivity patterns between the two methodologies.

**Results:**

We successfully partitioned signal activity into clusters of parcels that exhibit left-right symmetry between hemispheres and which align well with known anatomical regions of the dorsal surface of the pigeon brain. Moreover, functional connectivity matrices reveal positive correlations between homotopic regions. These cluster partitions and functional connectivity maps display similar patterns across and within individuals. Finally, WOI imaging results were comparable to resting state data acquired using fMRI.

**Conclusions:**

Taken together, these results demonstrate the potential of optical imaging technology for the reliable and cost-effective characterization of functional connectivity in birds. In addition, they position optical imaging methods as a valuable tool for large-scale comparative and network-level studies in this taxon.

## 1 Introduction

Recent advances in noninvasive functional neuroimaging have been instrumental in developing our understanding of how the brain integrates and processes information. For instance, cognitive processes and behaviors that were previously believed to be solely driven by specific brain regions are now recognized to be the result of coordinated neural networks that can involve diverse regions of the brain.^1^^,^^2^

Delineating the structure and organization of neural networks, along with the mechanisms that link their internal properties to variation in cognitive ability, is essential for a better understanding of how complex cognition emerges. One means to do so is examining the relationships of interconnected but anatomically separate neurons exhibiting synchronized patterns of spontaneous neurovascular activity^3^^,^^4^—in other words, examining resting state functional connectivity. A range of specialized tools, including functional magnetic resonance imaging (fMRI), electroencephalography (EEG), and magnetoencephalography (MEG), are currently available to characterize such correlations.

In humans, functional connectivity is primarily characterized using fMRI. Nevertheless, imaging non-human subjects using this system is limited by several critical constraints. For instance, MRI machines are costly to acquire and operate, nonportable, and they are restricted to organisms that can be studied in laboratories. Imaging small species requires specialized small-bore scanners with high magnetic field strengths to achieve sufficient spatial resolution for their small brains, making it challenging to compare data with large focal species.^3^^,^^4^ In addition, lengthy imaging sessions are often needed to improve spatial resolution due to lower signal-to-noise ratios associated with higher spatial resolutions, especially for small species.

As interest in non-model systems grows and the field strives to gain a more comprehensive understanding of cognition in the animal kingdom,^5^ the limitations of MRI technologies have become particularly constraining. To put this in context, of the ∼11,000 species of extant birds, less than 10 are well known to neuroscience.^6^^,^^7^ Without innovative solutions, expanding our knowledge base to encompass a wider range of species will likely remain an insurmountable challenge.

We propose optical imaging as an ideal technology to overcome some of fMRI’s constraints and to characterize the brain networks of birds. Optical imaging devices are significantly more affordable to produce and operate than MRI machines, and they can image a wide range of brain sizes without requiring any qualitative modifications of the technology. Optical intrinsic signal (OIS) imaging, a widefield optical imaging (WOI) technique, measures variations in the hemodynamic response to neural activity using reflected light. Specifically, when neurons fire, coupled neurovascular signaling initiates a rush of oxygen-rich blood to activated regions. Given that oxygenated and deoxygenated hemoglobin absorb light differently, local variation in reflected light intensity can therefore be used to track correlated changes in neural activity across the brain over time.^8^^,^^9^ In mice, although WOI was originally a niche technique, it has now become a mainstream tool and is embraced by high-profile neuroscience groups including the Allen Institute. It has been used to study a wide range of neuroscience topics, from brain parcellation to plasticity and across a number of diseases (e.g., stroke, Alzheimer’s, developmental disorders, to name a few).^10^^,^^11^ High-resolution WOI has now successfully imaged the entire cortex in mice^9^^,^^10^ and has been used with different indicators, optogenetics, head-fixed cognitive tasks, and freely moving behavior.^12^^–^^14^

Although WOI has become a powerful tool for neuroscience research in mice, its potential in avian species remains largely untapped. Birds and mammals diverged more than 300 million years ago and evolved distinct brain structures and cognitive adaptations, making birds exceptionally valuable for comparative studies. For instance, avian brains lack several canonical mammalian brain features, including a corpus callosum and a layered cortex.^15^ Because of these differences and their relatively small brain sizes, birds were once assumed to have limited cognitive capacities. However, contemporary evidence indicates that although the architecture differs, birds possess many structures that are functionally similar to the mammalian neocortex.^16^^–^^18^ Moreover, despite having a smaller overall brain size, birds possess brain structures that are functionally analogous to the mammalian neocortex and have neuron counts and cognitive abilities comparable to mammals, including primates.^19^^,^^20^ Similar to mice and primates, avian model organisms such as pigeons can perform head-fixed cognitive tasks and can be trained.^21^ In addition, pigeons and other bird species^22^ can be genetically encoded with calcium indicators (e.g., GCaMP6s delivered by virus),^23^ similar to existing mammalian models. Furthermore, because oscine songbirds, parrots, and hummingbirds learn their vocalizations, birds offer a powerful comparative system for studying vocal learning alongside mammalian models, including those relevant to human language acquisition.^24^^–^^27^ Despite this promise, only a handful of optical imaging studies exist in birds, and these have been limited to specific regions^28^ or low-resolution imaging^29^ during behavioral tasks.^21^^–^^23^

Here, we evaluate the suitability of optical imaging for assessing functional connectivity in birds by comparing WOI and fMRI in captive pigeons. WOI is ideal as a proof-of-concept because it offers high spatial resolution, rapid volume acquisition speeds, and a high signal-to-noise ratio.^30^ We begin by using iterative parcellation and hierarchical clustering to divide the dorsal surface of the telencephalon into discrete functional units and to show that these units collocate with known anatomical regions in the pigeon brain. In addition, we use well-established methods^10^ to estimate parcel relationships and demonstrate that the underlying patterns of functional connectivity within and across hemispheres are not only consistent across different imaging sessions for the same individual but also across sessions for different individuals. Finally, we show that our WOI results align with those obtained through fMRI imaging on a separate cohort of individuals.

## 2 Materials and Methods

### 2.1 WOI

#### 2.1.1 Animal preparation

All animals and procedures used in this section of our project were approved by the Washington University School of Medicine’s Animal Studies Committee via protocol # 22-0289. Four adult White Carneau and four adult homing pigeons (both breeds: *Columba livia*) were anesthetized with 80  mg/kg ketamine and 30  mg/kg xylazine in saline, through intramuscular injection into the pectoralis major. We verified anesthesia depth with toe pinch and blinking or pupil reflex, and once successfully induced, the animal was secured on a stereotaxic frame using ear and bite bars. Eye lubricant was applied to protect the eyes. We trimmed all feathers on the scalp to the skin line, and the scalp was retracted over the viewing window area following administration of 2% lidocaine topical anesthetic. Post scalp retraction, we fitted the imaging area with a 2.4  cm×4  cm×1.5  mm plexiglass viewing window and secured it to the skull using clear Metabond dental cement (C&B-Metabond, Parkell Inc, New York, USA). This window helps secure the pigeon in place at the correct angle during scanning while allowing light to pass through.

#### 2.1.2 Optical imaging system and image acquisition

**Fig. 1:**
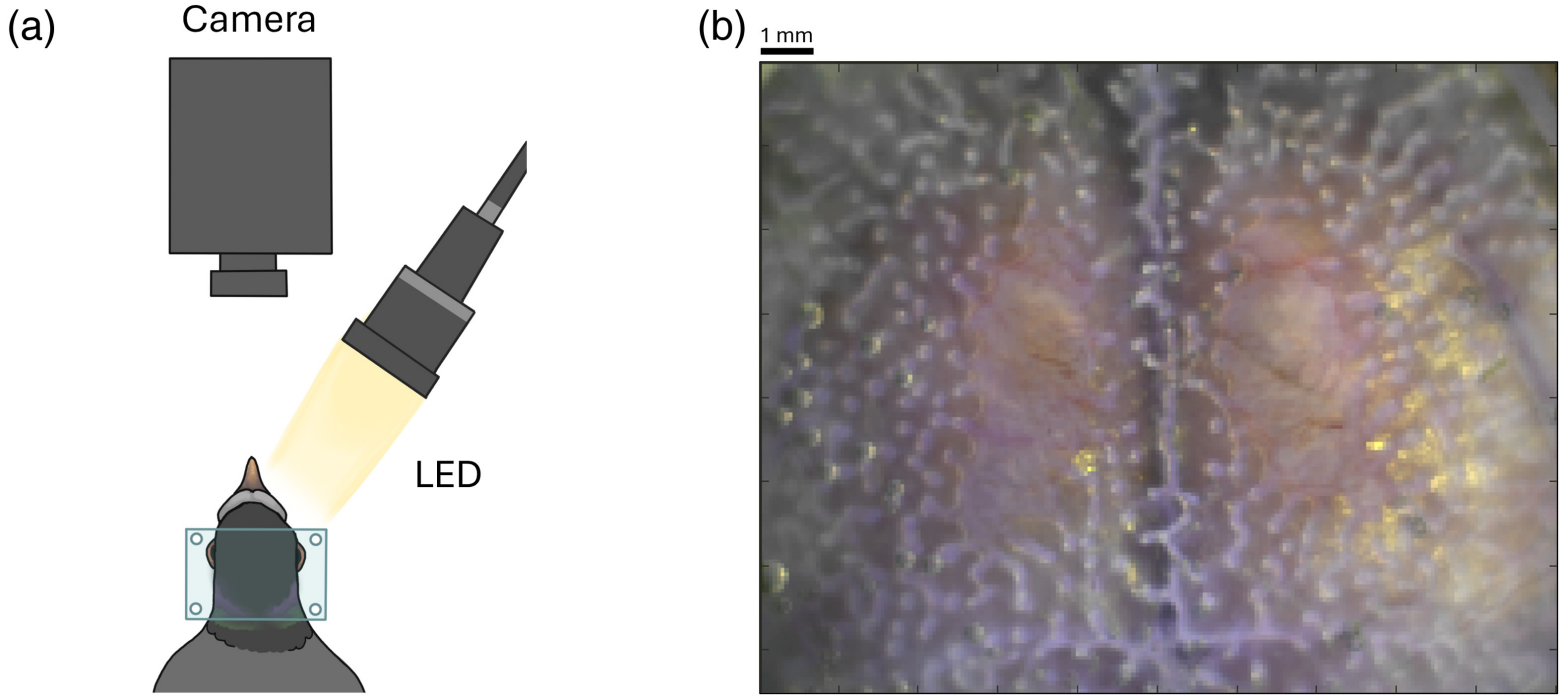
System setup for pigeon WOI. (a) Skull surface is illuminated by sequentially firing LEDs (530, 590, and 625 nm) sharing a collinear optical path using dichroic mirrors. Detection by a sCMOS camera at an image-capture frequency of 120 Hz (16 frames per second after light channel separation and before temporal down-sampling). (b) Reconstructed white light image of the surface of the pigeon’s skull as generated from our LED channels. The camera’s viewing window covered ca. 11  mm×15  mm, or 1200×1600  pixels. This window was sufficient to image most of the dorsal telencephalon, visible through the skull and centered over the visual Wulst region.

Although still under ketamine/xylazine anesthesia, study individuals were secured to a stereotaxic frame using the imaging window. The skull surface was illuminated through sequentially firing light-emitting diodes (LEDs, 530, 590, and 625 nm), which share a collinear optical path using dichroic mirrors. Reflected light was detected by a sCMOS camera (Zyla 5.5, Andor Technologies; Belfast, Northern Ireland, UK) at an image-capture frequency of 120 Hz (16 frames per second after light channel separation and before temporal down-sampling; Fig. 1(a). The LEDs and the camera were time-synchronized and controlled via computer using custom-written software (MATLAB, Mathworks). The camera viewing window (1200×1600  pixels, or roughly 11  mm×15  mm) was sufficient to image most of the dorsal telencephalon, visible through the skull and centered over the visual Wulst region [Fig. 1(b)]. Between 4 and 9 runs were collected in 5-min bouts for each individual while the pigeon was anesthetized; the total runs collected depended on the pigeon’s anesthesia depth.

#### 2.1.3 Pre-processing

After acquisition, all images were preprocessed in MATLAB (R2023a) using custom scripts. Images were spatially and temporally downsampled to 75×100  pixels and from 16 to 8 frames/second. The imaging window was manually masked to exclude non-brain regions, areas of signal fall-off, and out-of-focus edge sections. Mask boundaries were chosen using both a “white light” image of the skull generated from the first acquisition frame and from a map of the reshaped r values of every pixel against the averaged global signal. Temporal drift and spatial variation were corrected with second-order spatial and temporal detrending. Detrended data were log-mean transformed as the log of the ratio of the data to its temporal mean, $\log(\frac{\text{data}}{\langle \text{data}\rangle \;})$, resulting in a change in absorption relative to the mean. Differential absorption data were adjusted for wavelength-dependent pathlength factors,^10^ then converted into oxygenated and deoxygenated relative hemoglobin concentration contrasts (using hemoglobin extinction values from Ref. 31).

Differential hemoglobin contrast images were then spatially smoothed using a 5×5  pixel Gaussian filter with a standard deviation of 1.3 pixels. As the averaged signal in the left hemisphere differed from that of the right, the average hemispheric signal was regressed from each hemisphere, in lieu of global signal correction. To delineate the two hemispheres, the existing binary mask was divided into separate left and right masks using the natural visual division created by the longitudinal skull suture that joins left and right frontal bone plates (see Supplementary Material). Time traces of all pixels defined as brain within each hemisphere were then averaged to create a hemispheric signal that was regressed from its corresponding hemisphere to remove this source of noise. Finally, the signal was bandpass filtered to include only the frequency band between 0.02 and 0.167 Hz. This band was chosen because it includes the frequencies at which functional connectivity signals are likely to be strongest^32^ and matches the range of our collected fMRI data, which are limited to 0.167 Hz at the highest frequency due to limits to fMRI volume acquisition speed. 0.02 Hz was chosen as the lower bound because noise at lower frequencies was unable to be regressed out.

#### 2.1.4 Iterative parcellation and functional connectivity mapping

All runs were quality checked after processing. See the Supplementary Material for a flowchart of our processing pipeline. All runs from a given individual that passed quality controls were temporally concatenated. A final inspection of the averaged signal across all time points within the masked data was made, and time points with a sum of squared intensity value (i.e., the relative ${\mathrm{HbO}}_{2}$ concentration) above 1 were identified as possible sources of physiological or other noise and excluded from our analyses. Given that previous studies have shown similar results using either oxygenated or deoxygenated hemoglobin contrasts,^33^ we used only the ${\mathrm{HbO}}_{2}$ contrast data for our analysis.

Our goal was to partition pixels within the brain into component regions for correlation analysis. However, without a corresponding anatomical scan such as CT or MRI, we were only able to approximate the locations of the anatomical brain regions in our imaging window from features on the skull (see Supplementary Material). Accordingly, functional units within the brain were delineated using iterative parcellation following Ref. 10. For this parcellation method, the time traces of every brain pixel were first correlated against every other pixel using Pearson’s correlation to create a connectivity matrix. The connectivity information in this matrix was condensed using singular vector decomposition, hereafter SVD (“svd” built-in function, economy size decomposition, MATLAB), into a series of orthogonal vectors.

As these vectors are ordered by the relative contribution of each vector to the overall data variance, the first vectors represent the most dominant connectivity patterns across the brain and were used to assign all pixels to an initial parcel depending on the vector with which it was most strongly positively or negatively associated. From this initial parcellation, new time traces were generated that were an average of the time traces of all pixels within each parcel, and then, each pixel time trace was correlated with each new parcel average time trace. Using this new correlation matrix, each pixel was reassigned to either the same or a new parcel, depending on the maximum correlation value of the pixel to parcels. These new parcels were used to make new parcel average time traces, and each pixel’s correlation coefficient with all parcels was calculated again, until finally arriving at a solution where no additional parcel assignment changes occurred. We note here that the dorsal surface of the pigeon brain has a large vein (i.e., the dorsal longitudinal venous sinus^34^^,^^35^) that runs down the center of the scan between the left and right hemispheres and obscures the signal. Typically, the activity patterns from pixels along this vein strongly correlate with one or more vectors derived from SVD. Thus, to avoid possible biases in our analyses, we ran an initial SVD on the entire field of view, identified all highly positively correlated pixels assigned to parcels in this general area, and manually masked them out before running the SVD and iterative parcellation analysis once again to determine parcel assignments.

We estimate that the size and position of our imaging window should capture ∼10 functionally independent brain regions in the pigeon’s brain, given available information on anatomical^36^^,^^37^ and functional^38^ brain subdivisions in this model organism. We scanned individuals at two different angles, which captured slightly different portions of the dorsal surface of the telencephalon: one rostral and closer to the beak, and one caudal and closer to the cerebellum. In the caudal placement, scanning window boundaries were expected to contain the hippocampal formation, visual and somatosensory Wulst, a lateral portion of the mesopallium, and area corticoidea dorsolateralis (CDL). In individuals with a rostral window placement, the window covered less of the hippocampal formation and included the outer edge of the nidopallium (NFL, nidopallium frontolaterale).

Neurons within the same anatomical region of the brain are generally expected to have a homogenous signal due to shared function.^39^ However, larger regions that are anatomically distinct at a given spatial level can also often be functionally subdivided, and thus, areas within a larger-scale region may exhibit distinct variation in functional connectivity patterns (variation in function of distinct neural populations within the hippocampus, for instance).^40^^–^^43^ Given the scarcity of available information on how these anatomical regions might functionally subdivide when measuring spontaneous hemodynamic fluctuations in the pigeon, we assessed functional connectivity patterns in two steps. First, we used iterative parcellation following the methods outlined in Ref. 10 to subdivide our field of view into 30 parcels. We then performed a hierarchical clustering analysis via the clustergram function in MATLAB^44^ to group those parcels into hierarchical clusters based on the average trace similarities at each grouping level. Finally, we used the resulting dendrogram to identify the first grouping level that divided the field of view into at least eight clusters (range across all individuals: 8 to 12), which is the number of anatomical regions expected to be detected in our field of view, given the multiple pigeon brain atlases available.^36^^,^^37^

**Fig. 2:**
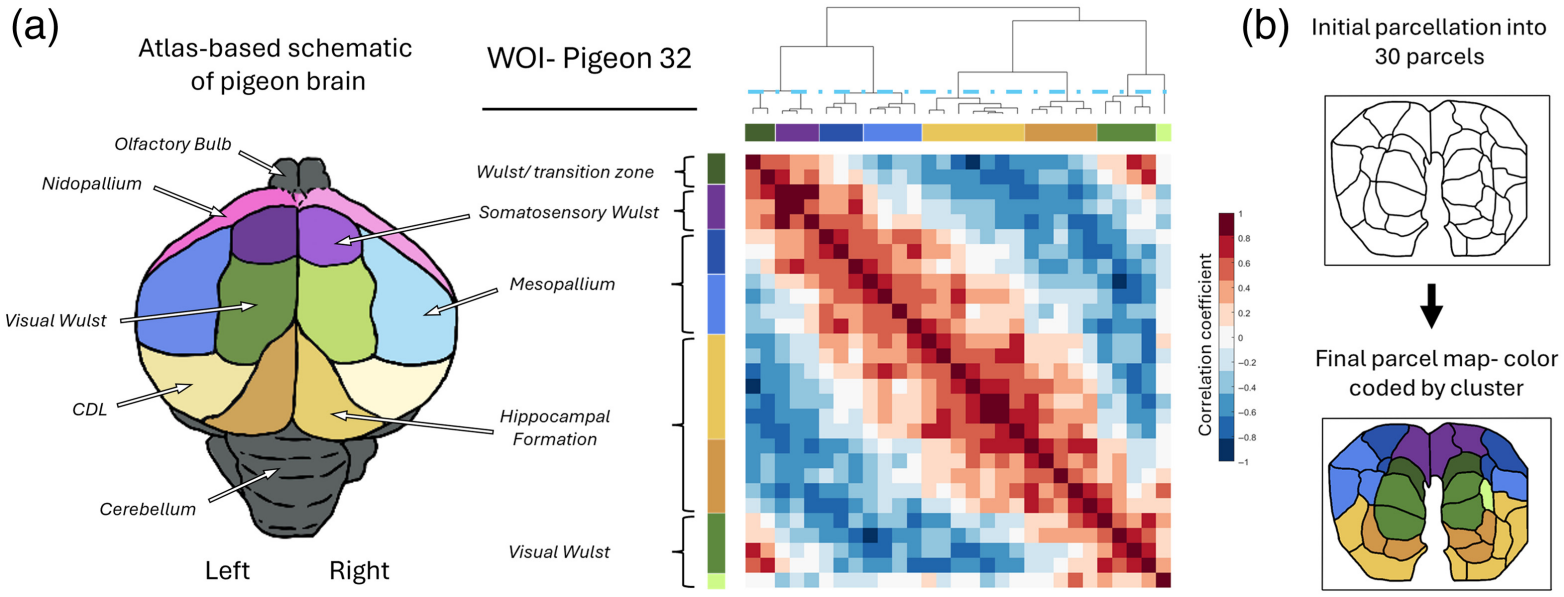
Functional organization of the dorsal surface of the pigeon’s brain. (a) Correlation matrix of activity patterns of 30 parcels extracted through iterative parcellation on WOI data from one test subject. The associated dendrogram depicts the results of a hierarchical clustering analysis based on observed correlation, and the blue dashed line depicts the dendrogram depth at which at least eight clusters are first observed. The resulting higher-level clusters are highlighted with color bars adjacent to the X and Y axes of the pairwise correlation matrix. Brain regions that coincide with each cluster (determined by comparing the centroid coordinates of a cluster of parcels with the location of known-brain regions in existing brain atlases^36^^,^^37^) are labeled to the left along with an atlas-based schematic of the brain regions visible within our viewing window. Similar colors are used to depict parcels located within the same anatomical region (e.g., green shades highlight parcels located within the visual Wulst). (b) Parcellation maps at different stages of analysis. The top map depicts the results of our initial iterative parcellation analysis, whereas the bottom map highlights the higher-level associations observed in our hierarchical clustering analysis (cluster colors match those in the color bars in the correlation map).

Pigeons are not as thoroughly studied as mice or humans, and only a partial MRI atlas currently exists for them.^37^ However, a complete histological atlas exists for the pigeon brain in sagittal and coronal view^36^ as well as many other resources including a detailed analysis of the pigeon connectome.^38^ These resources, as well as skull landmarks (S2 in the Supplementary Material) and expert consultation, were used to identify the known anatomical regions to which each cluster belongs [coded as colors in Fig. 2(a)].

### 2.2 fMRI

#### 2.2.1 Animal preparation

Ten Valencian Figurita breed adult pigeons (three females, seven males) were used to compare WOI imaging results with fMRI. The fMRI study complied with the National Institutes of Health Guidelines for laboratory animal care and was approved by the State of North Rhine-Westphalia ethics committee (LANUV, application no. Az.: 81-02.04.2021.A240). Study animals were kept individually in wire mesh cages ($45\times 45\times 45\;{\mathrm{cm}}^{3}$), under a 12-h light-dark cycle, and given *ad libitum* access to food and water. To prevent motion artifacts and to ensure pigeons remained calm during the awake scanning procedure, pigeons were surgically implanted with an MR-compatible pedestal and gradually habituated to the scanner environment and head-fixation with a custom-made restrainer.^21^^,^^45^ Briefly, study animals were secured in a restraining jacket and placed in a darkened room inside a custom mock scanner to expose them to the testing conditions. Exposure length began at 10 min duration and gradually increased to 120 min over a period of 2 weeks, with one habituation session per day. At the conclusion of this period, scanner noise was added to the habituation setup, and pigeons were further exposed to the scanner noise for 4 days before the beginning of the experiment.

#### 2.2.2 Image acquisition

All scans were acquired using a 7T small-bore horizontal MR scanner (Bruker BioSpec 70/30 USR, Ettlingen, Germany), equipped with a quadrature birdcage resonator (82 mm ID) as RF transmit coil and a single-loop 20-mm ring receiver coil, and using ParaVision 6.0.1 software to acquire image data. Study pigeons were secured using head fixation and custom restraint jackets and placed in the scanner with a pneumatic pillow (Small Animal Instruments, Model 1025 T monitoring and gating system) positioned under the wing on the chest to track respiration and ensure it stayed within acceptable parameters. Before beginning functional scans, scout images were taken using a multi-slice rapid acquisition sequence to locate the brain and slice positions.^31^ After measuring the map shim, a localized shim was performed for the whole brain.

After alignment and tuning, whole-brain resting-state images of study pigeons were acquired using a single-shot multi-slice gradient echo EPI sequence (GE-EPI) with the following parameters: ${\mathrm{TE}}_{\mathrm{eff}}$: 10.577 ms, TR: 1500 ms, 15 coronal slices with interleaved slice order, slice thickness of 0.8 mm with no slice gap, in-plane spatial resolution: $0.403\times 0.403\;{\mathrm{mm}}^{2}$, FOV: $25\times 25\;{\mathrm{mm}}^{2}$, and 800 repetitions. Four FOV saturation slices were placed over the eyes to avoid brain image corruption due to eye movements. The total scan time was 20 min. To check the reproducibility and stability of the results, rsfMRI data of all animals were recorded twice on different days. Following this scan, an additional 10 volumes were acquired with reverse phase encoding to correct for geometry distortions using FSL’s *topup* function. To improve registration of the functional images to the anatomical image, we acquired a T2-weighted in-plane TurboRARE anatomical image with the same slice dimensions as the GE-EPI sequence (spatial resolution: $0.403\times 0.403\;{\mathrm{mm}}^{2}$), followed by a 3D T2-weighted TurboRare anatomical image with ${\mathrm{TE}}_{\mathrm{eff}}$: 46.67 ms, TR of 1800 ms, RARE factor of 16, number of average: 2, spatial resolution: $0.2\times 0.2\times 0.4\;{\mathrm{mm}}^{3}$, and matrix size of 110×110×55 for spatial registration. Total time in the scanner for each study pigeon was under 1 h.

#### 2.2.3 Pre-processing

fMRI data were preprocessed using FSL^46^^–^^48^ (version 6.0.7.11), the Analysis of Functional NeuroImages (AFNI)^49^^,^^50^ (version 24.2.07 ‘Macrinus’), and ANTs^51^^,^^52^ (version 2.5.1 ‘Forelius’) software before subsequently moving to MATLAB (R2023a) for SVD analysis and parcellation using the above-outlined WOI processing procedures. DICOM images were converted to NIfTI format, and voxel sizes were upscaled 10x to align with settings designed for human fMRI software. Next, motion artifacts from head movement were calculated using framewise displacement to determine the number of volumes affected by motion artifacts; acquisitions that passed the threshold of 160 volumes above 0.9 mm (18% voxel dimensions) were excluded from analysis. In total, only 10 and 4 volumes from two different runs of two different individuals showed framewise displacement above 0.9 mm. Not a single run had to be excluded.

BOLD signals were de-spiked using the *3dDespike* function in AFNI to truncate signal spikes, and images were motion corrected using FSL’s *mcFLIRT*.^46^ Non-brain tissue was manually masked out in *FSLeyes*, and global brain signal, ventricle (IV) signal, and motion parameters were regressed out with FSL’s *fsl_regfilt*. Time series were smoothed with a Gaussian kernel of 0.8 mm using AFNI’s “3dBlurInMask,” global intensity was normalized with a grand mean of 10,000 for group analysis, and time series were band-pass filtered at a frequency range of 0.01 to 0.1 Hz using AFNI’s *3dTProject*. Data were top-up corrected to reduce distortions caused by the susceptibility-induced field and better match functional to structural images during registration, and the first and last ten volumes were cut from the time series to remove volumes affected by the thermal noise effect and edge effect from temporal filtering, respectively.

Following this, we linearly co-registered the functional data to the in-plane anatomical (*flirt*, 6 d.o.f, trilinear interpolation) and in-plane anatomical to high-resolution T2-weighted anatomical images (*flirt*, 12 d.o.f, trilinear interpolation), using affine linear registration. As the BOLD and in-plane anatomical images began at the very top edge of the brain, we first zero-padded images with two additional rows in the y dimension to add space and prevent interpolation errors with missing data during registration. Anatomical images were bias-field corrected prior as it improved the accuracy of the registration (ANTs, *n4BiasFieldCorrection*,^53^ 3D, convergence resolution iterations 50×50×50×50, 0.8 mm resolution). To align structures for group analysis, the high-resolution data were additionally co-registered to a population average standard space (*flirt*, 12 d.o.f, trilinear interpolation; *fnirt*, warp resolution $6\times 6\times 6\;{\mathrm{mm}}^{3}$, membrane_energy). A population-based template was generated using *antsMultivariateTemplateConstruction.sh* script.^51^

Transformation matrices were concatenated to reduce degradation from interpolating the functional images multiple times, and BOLD images were z-standardized and transformed directly to standard space for group analysis in MATLAB. Unlike our WOI dataset, our fMRI data were collected as three-dimensional whole-brain volumes. To restrict the functional data as much as possible to the same dimensions and depth as WOI, preprocessed fMRI data were further manually masked to an ∼2  mm band on the dorsal surface of the telencephalon. The value of the cutoff for our fMRI data is based on previous optical imaging work, which shows that the wavelengths used in this study penetrate ∼500  μm into brain tissue.^54^ Given our fMRI slice thickness of 0.8 mm, we therefore chose a cutoff of two slices (∼2  mm depth) to approximate this depth while minimizing fMRI digitization error. This surface mask was applied to the whole brain data, and subsequent SVD and parcellation were carried out only on voxels within this mask, using a modified version of the custom MATLAB script used on the WOI data. This modified script was updated slightly for compatibility with the 3D nature of the fMRI data; otherwise, it remained the same.

### 2.3 Statistical Analysis

Correlation between homotopic regions can sometimes be particularly strong, resulting in parcels that include pixels in both the left and right hemispheres. We addressed this issue by dividing our parcellation maps by hemisphere after hierarchical clustering analysis and manually reordering the parcel numbers within them to assign the hemisphere-specific cluster identities used in subsequent functional connectivity analyses (See Sec. 3.2 and Figs. S3C, S4, and S5 in the Supplementary Material.).

We assessed whether functional correlation patterns differed between individuals using randomization tests. For each major region (e.g., L Wulst, R Wulst, L Hippocampus, R Hippocampus, etc.), we computed the average trace and all pairwise inter-regional correlations. We then generated 1000 null correlation matrices per individual by randomly shuffling the original matrices. For each shuffled pair, we calculated the mean difference in corresponding correlations between a shuffled matrix for individual 1 and one for individual 2, yielding a null distribution of expected similarity (light blue histogram, Fig. 4). The probability of obtaining the observed difference by chance was subsequently estimated as the resulting quantile within this distribution (red line in Fig. 4) using the ECDF function in R (version 4.5.0). Quantile values <0.05 suggest that the observed differences were unlikely to arise by chance. We applied the same procedure to assess within-individual stability across imaging sessions. In that case, the computed deltas reflect differences in corresponding regional correlations between session 1 and session 2 for the same individual, allowing us to evaluate whether longitudinal changes exceeded those expected under the null distribution.

To determine if homotopic correlations were significantly different from non-homotopic correlations, we again used parcels combined into regions to compare at a cross-individual level. As these correlations were not normally distributed (“kstest” in MATLAB, R2023a), we used a Wilcoxon rank sum test to assess if average Pearson correlation values between homotopic and non-homotopic region pairs across all individuals were significantly different.

## 3 Results

### 3.1 Parcellation

A plausible starting point for parcellation is that the brain should be divided into the same number of larger anatomical regions that are observable within a given field of view. However, anatomical boundaries may not necessarily correspond to functional ones, as sub-populations of neurons within larger brain regions can exhibit variation in function.^43^^,^^55^^,^^56^ To address this issue, we partitioned the brain at two spatial scales^57^: an initial “finer scale” iterative parcellation, followed by a hierarchical clustering analysis that grouped the initial parcels into 8 to 12 higher-order clusters based on anatomical brain regions.^36^^,^^37^ These steps follow the approach used by the first resting state parcellation performed in mice.^10^ We color coded each parcel according to its hierarchical clustering assignment [Fig. 2(a)] and relate the resulting clusters to known anatomical structures in the corresponding regions of the avian brain (See methods Sec. 2.1.4). Moreover, we use slight variations of a single color to depict parcels that were assigned to different clusters but were co-located within the same brain regions (e.g., green shades highlight different clusters observed within the visual Wulst).

**Fig. 3:**
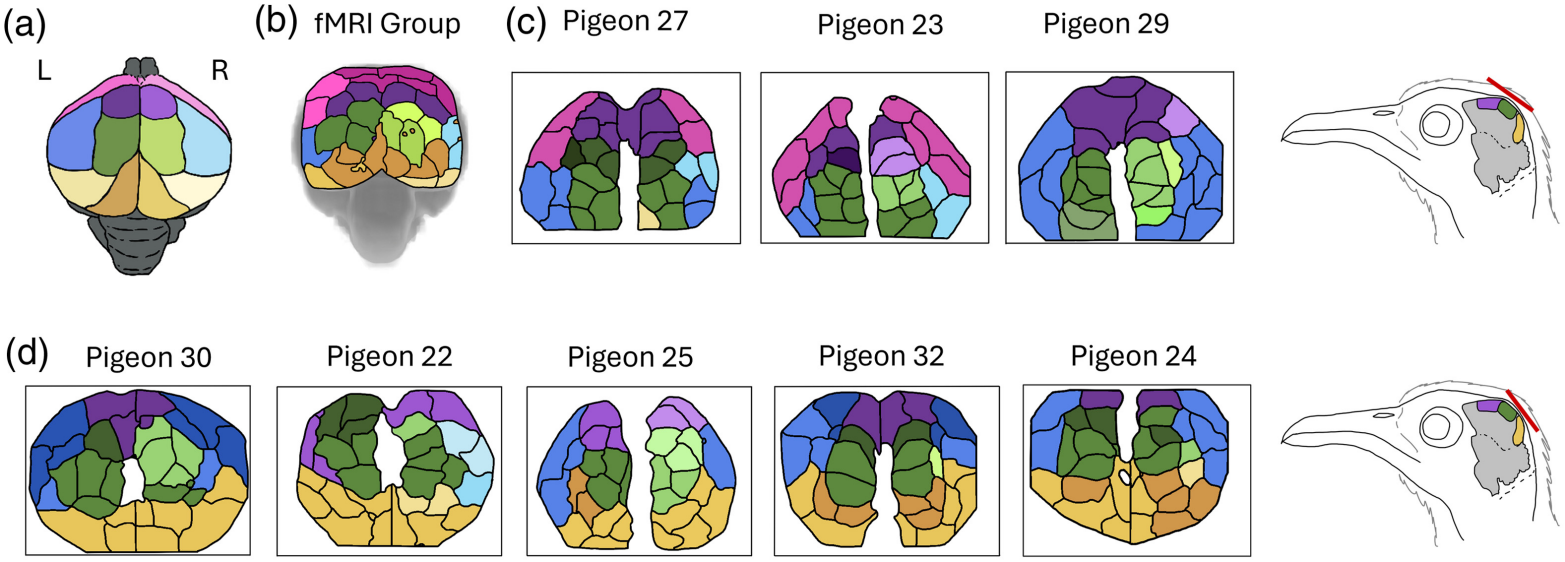
Variation in high-level parcel associations estimated from WOI and fMRI. (a) Each cluster is assigned a color based on anatomical region outlined in Fig. 2: somatosensory Wulst = purple; visual Wulst = green; mesopallium = blue; nidopallium = pink; hippocampal formation = yellow-orange. Clusters which were located only in the left hemisphere and clusters with parcels in both the left and right hemisphere received the color shown on the left side of the key or shades that are darker than the color shown in the left hemisphere in the key, while clusters located in only the right hemisphere received shades that were the same as the color shown in the right hemisphere of the key or lighter. Each color represents one cluster. (b) Cluster map of parcels from the fMRI dataset shown here for comparison. fMRI analysis is overlaid in this graphic on a top-down 3D surface view of the whole brain, angled to correspond with the viewing window of our WOI analysis. fMRI parcellation is derived here from a group-level analysis due to the lower signal strength of fMRI data. (c) High-level clusters and parcellation maps for different test subjects imaged with a WOI viewing window placed rostrally on the skull (window is depicted as a red line in the diagram to the right). (d) High-level clusters and parcellation maps for different test subjects imaged with a more caudally located WOI viewing window.

Using this parcellation and clustering method [Fig. 2(a)], we found high-level clusters that were symmetrically organized across the left and right hemispheres [Fig. 2(b)]. Moreover, resulting cluster shapes were consistent with anatomical organization in the pigeon brain [Fig. 2(b)], suggesting that our imaging tool and methods were successful at capturing anatomically relevant subdivisions within our viewing window. For instance, the green parcels in Fig. 2(b) overlap with the visual Wulst, a brain region that can be visually distinguished in our field of view as an oval shape located on the ridge of the medial side of both left and right hemispheres. Similarly, the hippocampal formation, which in birds is superficially located and curves around from the medial to lateral dorsal side of the left and right hemispheres, is largely coincident with the yellow-orange-colored clusters as estimated from either fMRI [Fig. 3(b)] or WOI [Figs. 2(b) and 3(d)) imaging sessions. We note here that the center of the hippocampal formation is not visible in many of our WOI individuals because we masked out all pixels covered by the central vein prior to the parcellation analysis.

The CDL, also known as the dorsolateral corticoid area, is a thin, superficial brain region within our viewing window [Fig. 2(a)] that did not readily separate into a distinct cluster in our hierarchical clustering analysis. Instead, parcels located on or near the expected location of the CDL exhibited higher-level clustering with the hippocampus in four of the five WOI individuals with a caudal imaging window. One possible reason for this finding is that the CDL plays an active role as a gateway between sensory and limbic areas, and serves as a direct structural and functional connection to DM and DL in the hippocampus.^42^^,^^58^^–^^62^

We consistently recovered resting state connectivity from our study pigeons. Broadly speaking, high-level cluster delineations were robust across individuals, as well as within individuals over time. We nevertheless note that non-trivial variation in parcel delimitations and higher-level clustering is observable within our WOI dataset. For instance, some individuals exhibit a single cluster within the somatosensory Wulst area, whereas others show two or more [Figs. 3(c) and 3(d)]. However, although areas are sometimes divided into multiple clusters, clusters are shaped as expected for the anatomical region. For instance, visual Wulst clusters generally stayed within the area of the visual Wulst and did not extend unexpectedly into the expected location of adjacent areas such as the lateral mesopallium.

Similarly, high-level clustering is consistent over time within individuals, but parcel delimitations and region subdivisions are noticeably different at different time points (Fig. S3 in the Supplementary Material). This variation might be a result of variation in anesthesia depth across the scanning session in our WOI individuals. Alternatively, it could reflect the short length of the individual runs used when making within-individual comparisons because each run was 5 min, which may not be sufficient for the network structure to stabilize.^63^ We note that this potential limitation could be easily improved in future work by, for example, scanning individuals for longer intervals.

To further validate optical brain imaging in our avian model, we compared our WOI results with those estimated with similar parcellation and clustering methods on a separate batch of pigeons that were imaged with fMRI [Fig. 3(b)]. We note here that intrinsic differences between fMRI and WOI led to unavoidable differences in processing, including the addition of correction of spatial distortions in fMRI resulting from inhomogeneities in the magnetic field and differences in image acquisition speed (fMRI being much slower than WOI), etc. Similarly, given the weaker signal strength of fMRI, we followed standing practices for that imaging modality and presented those results at the group level, after co-registration to a standard space. Likewise, because WOI images are in two dimensions, whereas fMRI images are in three, fMRI parcellation involved masking brain regions deeper than 2 mm and is presented here as a top-down surface view to facilitate visual comparison with WOI. Overall, the parcel delimitations, high-level clusters, and inter-hemispheric symmetry observed with fMRI mimic those seen with WOI.

### 3.2 Functional Connectivity

**Fig. 4:**
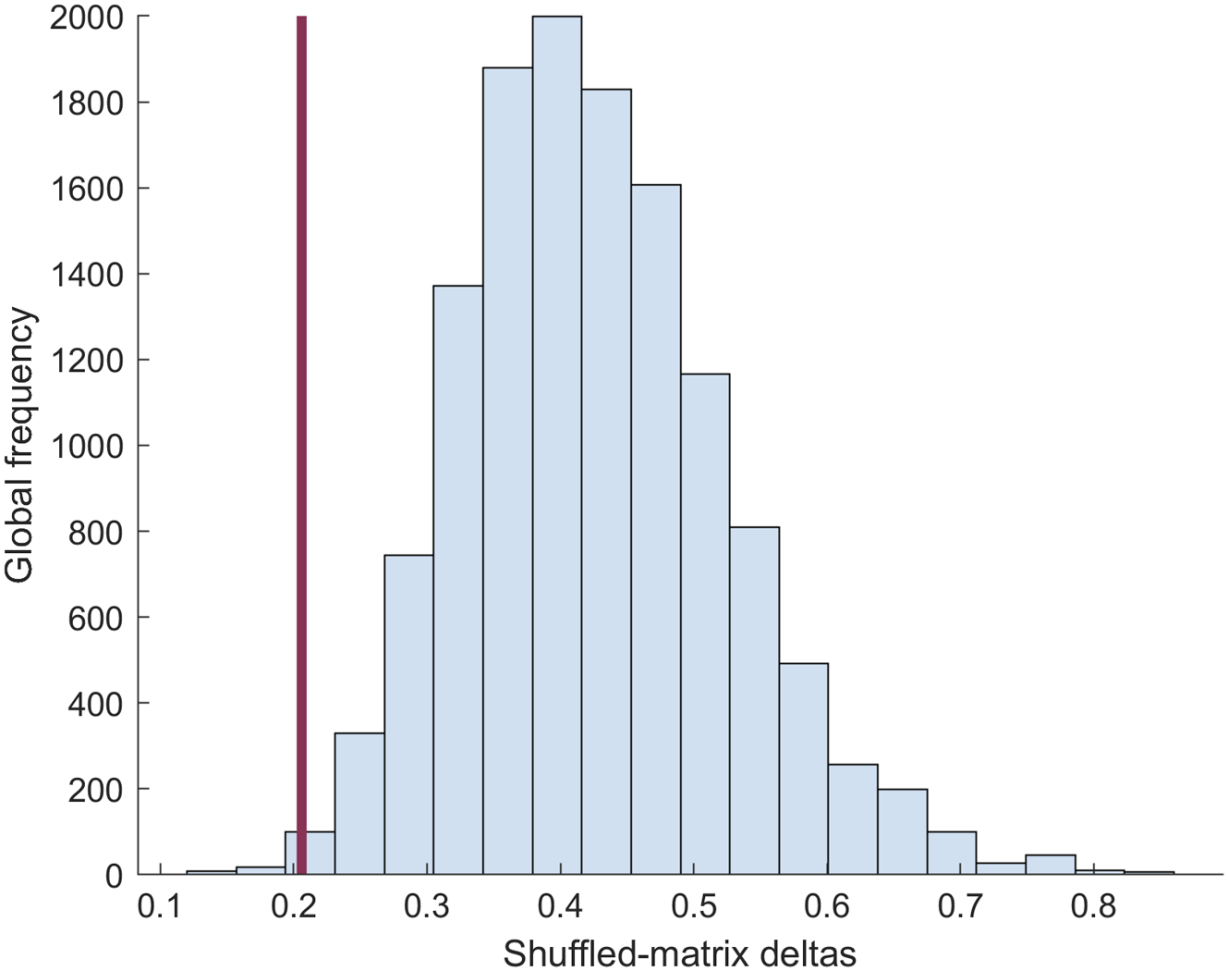
Comparison of mean observed matrix deltas (average value of all between-individual matrix deltas = 0.2092) against between-individual differences calculated after randomly shuffling the matrices. Comparisons were made between all individuals with rostral window placements and between all individuals with caudal window placements. The group average observed matrix delta is shown by the maroon line. To generate the shuffled matrix distribution (light blue histogram), each individual’s matrix was randomly shuffled, and then, the “between-individual” average was recalculated, 1000 times for each pair of individuals. Observed deltas were significantly smaller than would be expected by chance (probability of less than or equal to our observed value = 0.0016).

We performed a randomization test to determine whether functional correlation patterns were consistent across individuals. For each bird, we generated a correlation matrix using the average time series from each brain region within the imaging window. We then quantified between-individual differences by calculating pairwise dissimilarities among these matrices. To assess whether the observed mean difference exceeded what would be expected by chance, we constructed a null distribution by randomly shuffling the individual matrices and recomputing the between-individual differences (Fig. 4). The observed mean difference was significantly lower than the null expectation (P=0.0016, ECDF in R), indicating that individuals shared more similar correlation structure than would be expected by chance and demonstrating robust among-individual consistency.

**Fig. 5:**
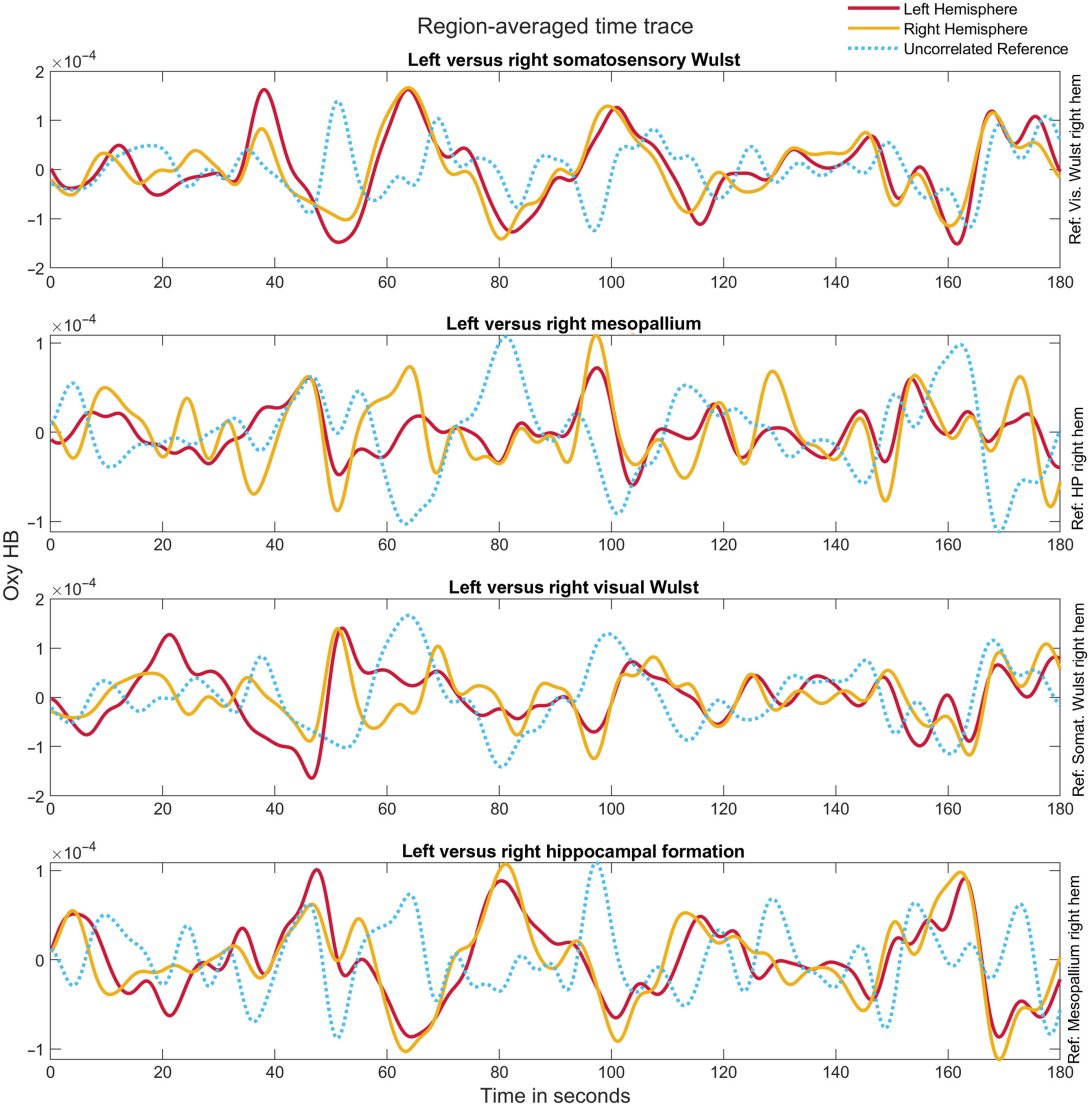
Correlation patterns in averaged activity from three selected regions in the pigeon brain. Average time traces in seconds (i.e., observed changes in oxygenated hemoglobin levels) for all pixels within four anatomical regions in the pigeon brain (Pigeon 32). For each time trace, the red line shows the average trace of the focal region in the left hemisphere, and the yellow line corresponds to the homotopic region in the right hemisphere. For comparison, an uncorrelated reference region is also shown (blue dotted line). Correlation values between each of the homotopic time traces are as follows: somatosensory Wulst, r=0.794; mesopallium, r=0.631; visual Wulst, r=0.607; and hippocampal formation, r=0.788.

We used a similar method to test variation in the underlying correlations within individuals, specifically by randomizing the correlation values across the first and second halves of different individuals. As in our initial test, we found that differences between the first and second halves of individual runs were significantly smaller than expected by chance (See Fig. S3 in the Supplementary Material), indicating that network structures are relatively stable while pigeons are in a resting state. In addition to general consistency across and within individuals, pigeons imaged with WOI exhibit bilaterally symmetric resting-state functional connectivity among parcels. For example, we observe positive Pearson correlations between the average signal within a region and the average signal within the region located in the same location on the opposite hemisphere (Fig. 5). These homotopic correlations are present across multiple spatial scales [At the region (Fig. 5)and cluster level and also at the parcel level (See Fig. S5 in the Supplementary Material)].

**Fig. 6:**
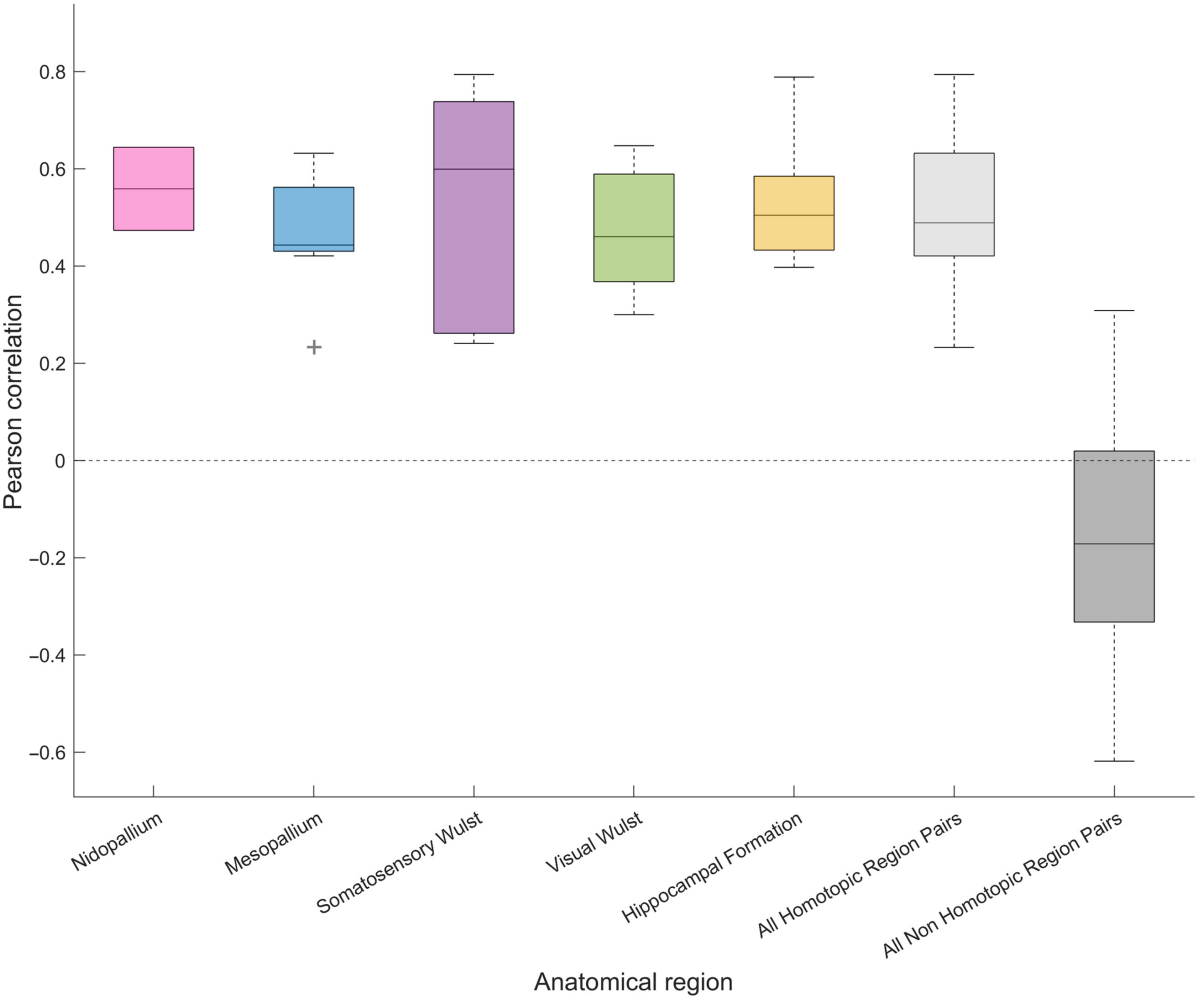
Comparison of Pearson correlation strength between homotopic and non-homotopic regions. The first five boxplots show the correlation between the left and right hemispheres for all large-scale brain regions within our viewing window (nidopallium, mesopallium, somatosensory Wulst, visual Wulst, and hippocampal formation) across all WOI individuals. Due to variation in viewing window placement, there is variation in sample size between regions (nidopallium: N=2; mesopallium: N=7; somatosensory Wulst: N=8; visual Wulst: N=8; hippocampal formation: N=5). Also shown is the distribution for all homotopic correlations together (N=30), as well as all non-homotopic correlations between regions (N=84) for all WOI individuals.

We found that these homotopic correlations are robust across individuals and represent the strongest positive correlations within our imaging window, differing significantly from non-homotopic correlations [Wilcoxon rank sum: P<0.001, z=−8.04; Fig. 6]. The median value of Pearson correlations for all homotopic region pairs was 0.49, compared with a median value of −0.17 for all non-homotopic region pairs. Correlations between non-homotopic parcels, clusters, and regions ranged from weakly positive to negative (e.g., visual Wulst and hippocampus). We note, however, that these negative correlations must be interpreted with caution because artificially strong anticorrelation is often introduced by removing the global signal during processing.^64^

**Fig. 7:**
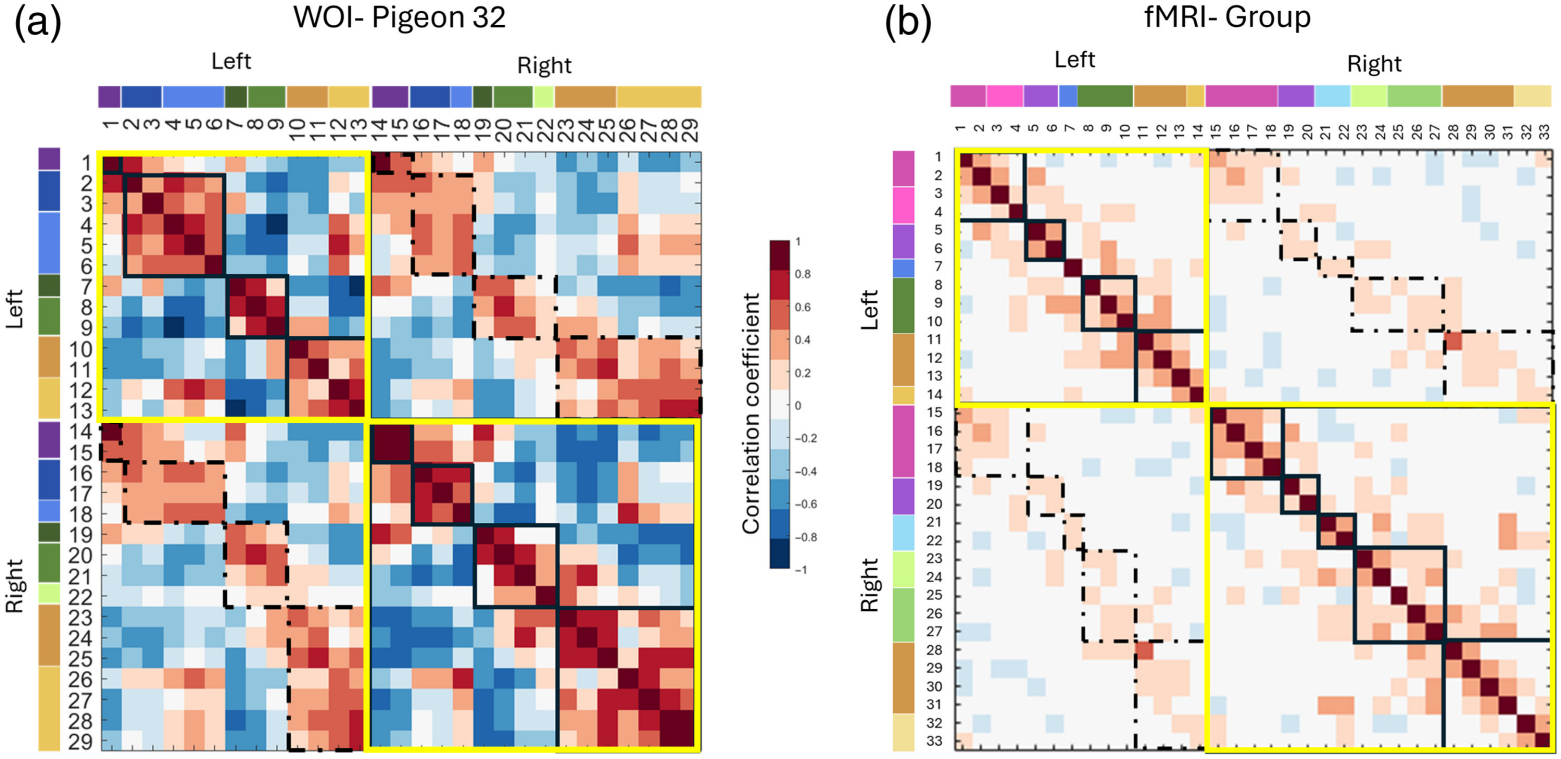
Patterns of parcel and high-level cluster correlation as estimated from WOI and fMRI data. Correlation matrices for (a) WOI and (b) group fMRI after dividing and re-ordering parcels by hemisphere and cluster identity (please note that the fMRI plot is based on a slightly larger viewing window that includes a region that was not imaged with the WOI individual shown). Cluster color assignments for each parcel are shown in bars at the left and top of the matrix next to parcel numbers. Data from within a single hemisphere (top left and bottom right) are highlighted with yellow squares, whereas data from high-level clusters in the same anatomical region are highlighted with smaller black squares. Lower left and upper right quadrants in these matrices depict cross-hemisphere correlations; homotopic comparisons within them are highlighted with dashed rectangles. Relationships above the diagonal are identical to those below it. Overall, all clusters exhibit strong homotopic correlation regardless of the imaging modality, although we note that these associations are noticeably stronger when estimated with WOI (both matrices use the same color scale).

**Fig. 8:**
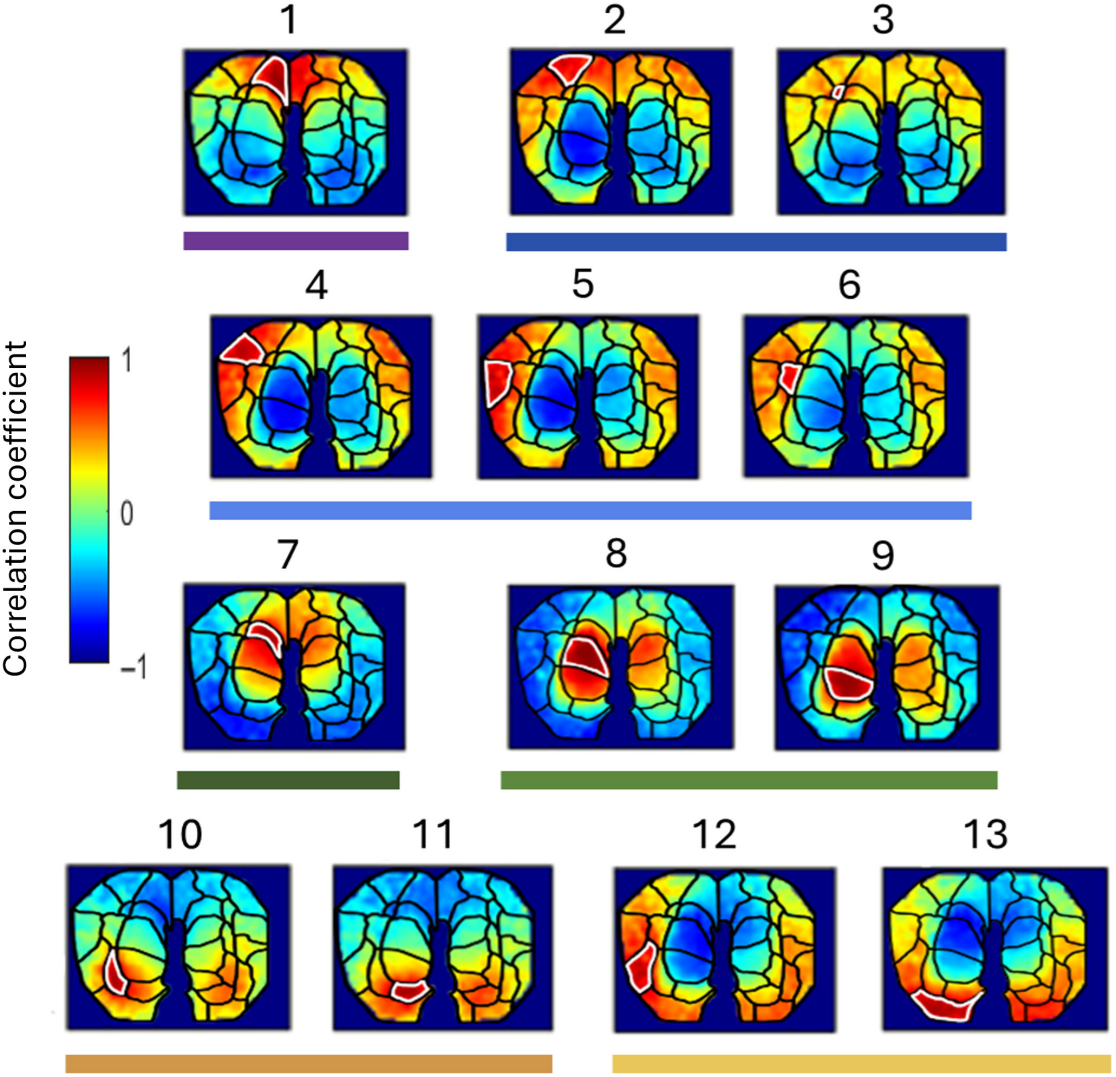
Correlation maps for all left hemisphere parcels in one of our WOI subjects (pigeon 32). An example presented as a supplemental way of visualizing these data that highlights parcel-level variation in correlation structure. To generate these correlation maps, the averaged time trace (oxygenated relative hemoglobin concentration contrast) of all pixels within the parcel is correlated against every pixel in the masked viewing window. Each focal parcel used for comparison in each subplot is highlighted with a white outline. Colors depict the strength of correlation; warm colors indicate a positive correlation between a pixel and the parcel average trace. These correlation maps are derived from the same analysis as the correlation matrix in Fig. 7(a), and parcel numbers above each map are thus equivalent to parcel numbers in Fig. 7(a).

These homotopic correlations were also found in our fMRI dataset [Fig. 7(b)]. When parcels are reordered by clusters within hemispheres [See Fig. 7(a) and Fig. S4 in the Supplementary Material for examples across all subjects imaged in this study], parcels within a cluster usually all positively correlate with one another, as well as positively correlating with their homotopic equivalents. This could be seen as evidence for dividing the brain into fewer parcels during initial iterative parcellation (i.e., the 8 to 12 selected for our hierarchical clustering analysis based on anatomical regions). However, we note that parcel relationships within high-level clusters are still not homogeneous, and there is variation among parcel relationships. This can be most obviously visualized in the pixel-level correlation maps in Fig. 8 (also see example time traces at the parcel level; Fig. S5 in the Supplementary Material), whereas there are generally positive correlations within a cluster, there is a fair amount of variation between parcels in the location of the strongest positive correlation areas in the opposite hemisphere. This parcel-level variation points toward potential sub-functionalization within larger brain regions.

## 4 Discussion and Conclusion

We have shown here that optical imaging technology is a suitable method for characterizing functional connectivity in an avian model system. Through wide-field optical imaging, we successfully imaged scalp-retracted anesthetized pigeons and recovered the basic hierarchy of association between brain parcels in the field of view. The observed cluster boundaries exhibit strong symmetry between hemispheres and align well with known anatomical regions of the dorsal surface of the pigeon’s brain. Moreover, the functional connectivity matrices estimated from these parcels include significant positive correlation between areas located in homotopic contralateral clusters, at parcel, cluster, and region spatial scales. Finally, the estimated cluster partitions and functional connectivity maps are similar within and across individuals, as well as between WOI and fMRI datasets. Collectively, these results suggest that optical imaging of hemodynamics is a highly effective and cost-efficient tool for characterizing network organization and functional connectivity in birds.

Although we followed parcellation methods used for mice, a direct comparison of the accuracy of our parcellation with parcellation results found in mice^10^^,^^11^ may not be possible, as the level of detail is not comparable between these species’ respective MRI atlases.^37^ Furthermore, at a conceptual level, avian forebrain topography and topology are much different from mammals, and the connectivity patterns underlying parcellation and brain structure are vastly different from those of mammals. For instance, bilaterally symmetric positive correlations across homotopic regions are commonly observed in humans and other mammals.^65^ However, because birds lack a corpus callosum, the extent to which these patterns should be expected in avian models is uncertain. fMRI studies in pigeons comparing the right and left hippocampus^42^ and entopallium^21^ have provided some evidence supporting the existence of such positive relationships. There is also some preliminary evidence of homotopic correlations in the song system of the zebra finch.^66^ The robustness of the homotopic correlations found in our study (Figs. 6 and 7 and Fig. S4 in the Supplementary Material) further supports the idea that homotopic correlations are a shared feature between mammals and birds. However, with fewer commissures and neurons connecting the hemispheres in birds, it is an open question whether information transfer is as fine-grained as in mammals, despite these similar hemodynamic correlations.

The functional connectivity maps of the more fine-scaled parcels within a cluster were often similar but notably not identical. In fact, some parcels produced substantially different functional connectivity maps despite being neighbors and collocating with the same anatomical region (for example, parcel 10 and parcel 11 in Fig. 8). Such differences could be partially artefactual, as spatial smoothing can differentially affect boundary pixels, particularly when they are located between two different high-level clusters. Further, the parcellation will be sensitive to the ratio of signal-to-noise in the data, where the noise may be from motion or confounding physiology.^39^^,^^67^ Alternatively, finer-grain parcellation may reveal true variation in correlation structure resulting from subfunctionalization of larger brain regions. An important point to consider is that both of these parcellation levels used in this study can be technically correct, but they offer us different perspectives across levels of organization within the brain.^57^ Avian hemispheres primarily communicate via the small anterior commissure that interconnects subcomponents of the amygdala and the arcopallium.^68^ Our results make it likely that area-specific alignments or misalignments of activity patterns are constituted by this core interhemispheric network. Future comparative studies may benefit from following similar approaches to understand how variations in interhemispheric coupling emerge in a class of vertebrates that was thought to be “natural split-brains”.

Another consideration is that manually choosing a preset parcel number presents a problem when the study species is not well-known to neuroscience. Pigeons are a model organism in avian research, and their neuroanatomy is relatively well-understood. However, many other avian species remain less well-known, necessitating an unsupervised approach to determining an ideal parcel or cluster number. Various alternative parcellation methods exist,^39^^,^^69^^,^^70^ but there is currently no universally accepted best practice. Parcellation methodology is an ongoing area of research, and a key goal of future work will be to develop methods suitable for intra- and particularly interspecific comparisons of avian functional connectivity networks. In particular, as functional connectivity can vary within an individual across brain states, it is perhaps more useful to understand these constructs as fluid entities that vary in shape and connectivity over time and should therefore be studied with more flexible methods.^71^

We chose to use WOI due to its high resolution, fast volume acquisition speed, and strong signal-to-noise ratio. In fact, our WOI results demonstrated stronger correlations than our fMRI results, allowing for individual-level comparisons. However, an important note is that differences between our processing methods for fMRI and WOI may also contribute to the variation in correlation strength seen in our study. Our WOI individuals needed to be anesthetized during imaging, whereas the fMRI individuals were habituated to the scanner and awake. Correlation strength could thus be influenced either directly due to the effects of anesthesia (which may vary based on anesthesia method, depth, and species^72^^,^^73^) or due to increased noise from the motion of awake birds (although note that fMRI processing included head fixation, framewise displacement calculation, and motion correction—see Sec. 2.2.1). In addition, our WOI dataset required separate hemispheric regressions due to differences between hemispheres, whereas global signal regression was sufficient for the fMRI individuals.

Although we have established that WOI is an exceptional tool for imaging the avian brain, we acknowledge that it still bears some potential limitations. For example, the technique used in this study (i.e., intrinsic signal optical imaging) is relatively slow, although we note that this issue can be addressed by incorporating pigeons (or other bird species) with a calcium indicator.^22^^,^^23^ Furthermore, many of these limits are not applicable to more advanced optical imaging techniques such as diffuse optical tomography (DOT). A significant constraint of WOI is the two-dimensional nature, which restricts imaging to the brain’s surface. Avian brains lack the separation between white and gray matter seen in mammals and exhibit instead a more nuclear organization with important functional regions distributed throughout the telencephalon.^38^ Thus, three-dimensional optical imaging alternatives such as DOT^33^^,^^74^^,^^75^ will be particularly useful for the characterization of more complete functional connectivity networks in birds.

Yet another important limitation of WOI is that it is strongly affected by obstacles in the field of view. In this study, for example, we were unable to observe brain activity under a large superficial vein in the center of the pigeon’s skull (dorsal longitudinal venous sinus), running along the suture between the two frontal plates.^16^^–^^18^^,^^34^^,^^35^^,^^76^ Here too, DOT offers a potential solution. By employing multiple source-detector pairs arranged in a grid across the skull, DOT can image around obstacles with comparable resolution to fMRI.^74^^,^^77^^,^^78^ Although DOT has been most commonly used in human studies, it has also been employed in non-human models such as non-human primates^79^ and mice.^80^ Recent efforts in DOT instrumentation have developed wearable, high-resolution systems, resulting in methods that are ultraportable, enabling field applications^74^ that do not require anesthesia or prior habituation coupled with surgery to enable head-fixation^81^^–^^85^ as is currently necessary for awake imaging of pigeons using fMRI.^81^^–^^85^ A final limitation of WOI is that, similar to fMRI, it currently requires exposing the skull and optionally installing a Plexiglas window. These invasive and time-consuming procedures are yet again, not necessary in DOT.

Despite these limitations, we believe that WOI, as is, can still be a valuable tool for imaging avian brains in a range of contexts. Unlike fMRI, WOI does not require positioning animals within a scanner bore, thereby avoiding many practical constraints associated with magnetic resonance imaging. WOI also provides spatial resolution comparable to, and in some cases exceeding, that of fMRI. Moreover, although its field of view is restricted, the superficial brain structures accessible to WOI are still highly informative for many research questions. For example, the avian hippocampus and the avian Wulst lie in the dorsal brain surface and are readily imaged with WOI as demonstrated here. Given that pigeons and many other bird species display advanced spatial navigation abilities and that taxa like corvids and parrots exhibit cognitive capacities comparable to those of primates,^19^^,^^20^ WOI holds considerable promise for future studies of learning, memory, and spatial navigation. WOI could also be used to capture stimulus-evoked activity in birds. For example, stimulation of the lateral visual field, which uses the thalamofugal visual pathway terminating in the visual Wulst (thought to be analogous to the primary visual cortex in mammals), would be expected to elicit a response within the same imaging window used in this study. Similarly, tactile stimuli could be used to demonstrate a response in the somatosensory Wulst.

In summary, we have demonstrated that optical imaging methods offer a viable and cost-effective alternative for studying functional connectivity in birds. Specifically, by imaging with WOI, we have proven here that optical imaging tools are capable of generating data of comparable quality and resolution to fMRI, at a fraction of the cost. Thus, optical methods hold promise to facilitate neuroimaging in non-model species and open the door to broader comparative studies on the evolution of cognition. Fully realizing this potential will nevertheless require overcoming current limitations, including the restricted depth and field of view inherent in 2D approaches. In that regard, advancing toward 3D imaging modalities such as diffuse optical tomography will be critical for capturing more complete neural networks and for developing noninvasive, portable systems suitable for awake and even field-based studies.

## Supplementary Material

10.1117/1.NPh.13.1.015010.s01

## Data Availability

Code for processing the fMRI data can be found at https://github.com/mehdibehroozi/sleeping_pigeon_fMRI. Code for processing the WOI data is modified from the following free toolbox (https://github.com/brierl/Mouse_WOI) described in Ref. 86. Custom code for iterative parcellation following Ref. 10 is available upon reasonable request. The data presented in this study may be obtained from the corresponding author upon reasonable request.

## References

[1] Bressler S. L. Menon V., “Large-scale brain networks in cognition: emerging methods and principles,” Trends Cognit. Sci. 14(6), 277–290 (2010). 10.1016/j.tics.2010.04.004 20493761

[2] Fox M. D. et al., “The human brain is intrinsically organized into dynamic, anticorrelated functional networks,” Proc. Natl. Acad. Sci. U. S. A. 102(27), 9673–9678 (2005). 10.1073/pnas.0504136102 15976020 PMC1157105

[3] Gebre R. K. et al., “Cross–scanner harmonization methods for structural MRI may need further work: a comparison study,” NeuroImage 269, 119912 (2023). NEIMEF 1053-8119 10.1016/j.neuroimage.2023.119912 36731814 PMC10170652

[4] Tax C. M. et al., “Cross-scanner and cross-protocol diffusion MRI data harmonisation: a benchmark database and evaluation of algorithms,” NeuroImage 195, 285–299 (2019). NEIMEF 1053-8119 10.1016/j.neuroimage.2019.01.077 30716459 PMC6556555

[5] Gogola J. V. et al., “NSF workshop report: exploring measurements and interpretations of intelligent behaviors across animal model systems,” J. Comp. Neurol. 533(3), e70035 (2025). JCNEAM 0021-9967 10.1002/cne.70035 40038068 PMC11879920

[6] Clayton N. S. Emery N. J., “Avian models for human cognitive neuroscience: a proposal,” Neuron 86(6), 1330–1342 (2015). NERNET 0896-6273 10.1016/j.neuron.2015.04.024 26087161

[7] Romanova E. V. Sweedler J. V., “Animal model systems in neuroscience,” ACS Chem. Neurosci. 9(8), 1869–1870 (2018). 10.1021/acschemneuro.8b00380 30107742

[8] Mayhew J. et al., “Spectroscopic analysis of neural activity in brain: increased oxygen consumption following activation of barrel cortex,” NeuroImage 12(6), 664–675 (2000). NEIMEF 1053-8119 10.1006/nimg.2000.0656 11112398

[9] Bauer A. Q. et al., “Effective connectivity measured using optogenetically evoked hemodynamic signals exhibits topography distinct from resting state functional connectivity in the mouse,” Cereb. Cortex 28(1), 370–386 (2018). 53OPAV 1047-3211 10.1093/cercor/bhx298 29136125 PMC6057523

[10] White B. R. et al., “Imaging of functional connectivity in the mouse brain,” PLoS One 6(1), e16322 (2011). POLNCL 1932-6203 10.1371/journal.pone.0016322 21283729 PMC3024435

[11] Benisty H. et al., “Rapid fluctuations in functional connectivity of cortical networks encode spontaneous behavior,” Nat. Neurosci. 27(1), 148–158 (2024). NANEFN 1097-6256 10.1038/s41593-023-01498-y 38036743 PMC11316935

[12] Pinto L. et al., “Task-dependent changes in the large-scale dynamics and necessity of cortical regions,” Neuron 104(4), 810–824.e9 (2019). NERNET 0896-6273 10.1016/j.neuron.2019.08.025 31564591 PMC7036751

[r13] Gallero-Salas Y. et al., “Sensory and behavioral components of neocortical signal flow in discrimination tasks with short-term memory,” Neuron 109(1), 135–148.e6 (2021). NERNET 0896-6273 10.1016/j.neuron.2020.10.017 33159842

[14] Gilad A., “Wide-field imaging in behaving mice as a tool to study cognitive function,” Neurophotonics 11(03), 033404 (2024). 10.1117/1.NPh.11.3.033404 38384657 PMC10879934

[15] Güntürkün O. Bugnyar T., “Cognition without cortex,” Trends Cognit. Sci. 20(4), 291–303 (2016). 10.1016/j.tics.2016.02.001 26944218

[16] Reiner A. et al., “Revised nomenclature for avian telencephalon and some related brainstem nuclei,” J. Comp. Neurol. 473(3), 377–414 (2004). JCNEAM 0021-9967 10.1002/cne.20118 15116397 PMC2518311

[r17] Jarvis E. D. et al., “Avian brains and a new understanding of vertebrate brain evolution,” Nat. Rev. Neurosci. 6(2), 151–159 (2005). NRNAAN 1471-003X 10.1038/nrn1606 15685220 PMC2507884

[18] Jarvis E. D. et al., “Global view of the functional molecular organization of the avian cerebrum: mirror images and functional columns,” J. Comp. Neurol. 521(16), 3614–3665 (2013). JCNEAM 0021-9967 10.1002/cne.23404 23818122 PMC4145244

[19] Emery N. J., “Cognitive ornithology: the evolution of avian intelligence,” Philos. Trans. R. Soc. B Biol. Sci. 361(1465), 23–43 (2006). 10.1098/rstb.2005.1736 PMC1626540 16553307

[20] Olkowicz S. et al., “Birds have primate-like numbers of neurons in the forebrain,” Proc. Natl. Acad. Sci. U. S. A. 113(26), 7255–7260 (2016). 10.1073/pnas.1517131113 27298365 PMC4932926

[21] Behroozi M. et al., “Event-related functional MRI of awake behaving pigeons at 7T,” Nat. Commun. 11(1), 4715 (2020). NCAOBW 2041-1723 10.1038/s41467-020-18437-1 32948772 PMC7501281

[r22] Katlowitz K. A. Picardo M. A. Long M. A., “Stable sequential activity underlying the maintenance of a precisely executed skilled behavior,” Neuron 98(6), 1133–1140.e3 (2018). NERNET 0896-6273 10.1016/j.neuron.2018.05.017 29861283 PMC6094941

[23] Nimpf S. et al., “Long-term, high-resolution in vivo calcium imaging in pigeons,” Cell Rep. Methods 4(2), 100711 (2024). 10.1016/j.crmeth.2024.100711 38382523 PMC10921020

[24] Brainard M. S. Doupe A. J., “Auditory feedback in learning and maintenance of vocal behaviour,” Nat. Rev. Neurosci. 1(1), 31–40 (2000). NRNAAN 1471-003X 10.1038/35036205 11252766

[r25] Jarvis E. D., “Learned birdsong and the neurobiology of human language,” Ann. N. Y. Acad. Sci. 1016(1), 749–777 (2004). ANYAA9 0077-8923 10.1196/annals.1298.038 15313804 PMC2485240

[r26] Miller J. E. et al., “Birdsong decreases protein levels of FoxP2, a molecule required for human speech,” J. Neurophysiol. 100(4), 2015–2025 (2008). JONEA4 0022-3077 10.1152/jn.90415.2008 18701760 PMC2576221

[27] Mello C. V., “The Zebra Finch, *Taeniopygia guttata*: an avian model for investigating the neurobiological basis of vocal learning,” Cold Spring Harb. Protoc. 2014(12), pdb.emo084574 (2014). 10.1101/pdb.emo084574 PMC4571486 25342070

[28] Keary N. et al., “Optical imaging of retinotopic maps in a small songbird, the zebra finch,” PLoS One 5(8), e11912 (2010). POLNCL 1932-6203 10.1371/journal.pone.0011912 20694137 PMC2915911

[29] Lee J. V. et al., “Noninvasive diffusive optical imaging of the auditory response to birdsong in the zebra finch,” J. Comp. Physiol. A 199(3), 227–238 (2013). JCPADN 0340-7594 10.1007/s00359-012-0788-0 PMC4371729 23322445

[30] Reisman M. D. et al., “Structured illumination diffuse optical tomography for noninvasive functional neuroimaging in mice,” Neurophotonics 4(2), 021102 (2017). 10.1117/1.NPh.4.2.021102 28439519 PMC5391480

[31] Prahl S., Optical Absorption of Hemoglobin, Oregon Medical Laser Center (2002).

[32] Cordes D. et al., “Frequencies contributing to functional connectivity in the cerebral cortex in ‘Resting-state’ data”. PMC7975218 11498421

[33] White B. R. et al., “Resting-state functional connectivity in the human brain revealed with diffuse optical tomography,” NeuroImage 47(1), 148–156 (2009). NEIMEF 1053-8119 10.1016/j.neuroimage.2009.03.058 19344773 PMC2699418

[34] Porter W. R. Witmer L. M., “Avian cephalic vascular anatomy, sites of thermal exchange, and the rete ophthalmicum,” Anat. Rec. 299(11), 1461–1486 (2016). ANREAK 0003-276X 10.1002/ar.23375 27258923

[35] Richards S. A., “Anatomy of the veins of the head in the domestic fowl,” J. Zool. 154(2), 223–234 (1968). JZOOAE 0952-8369 10.1111/j.1469-7998.1968.tb01660.x

[36] Karten H. J. Hodos W., A Stereotaxic Atlas of the Brain of the Pigeon: Columba livia, Johns Hopkins Press, United States (1967).

[37] Güntürkün O. et al., “A 3-dimensional digital atlas of the ascending sensory and the descending motor systems in the pigeon brain,” Brain Struct. Funct. 218(1), 269–281 (2013). 10.1007/s00429-012-0400-y 22367250

[38] Shanahan M. et al., “Large-scale network organization in the avian forebrain: a connectivity matrix and theoretical analysis,” Front. Comput. Neurosci. 7, 89 (2013).1662-5188 10.3389/fncom.2013.00089 23847525 PMC3701877

[39] Gordon E. M. et al., “Generation and evaluation of a cortical area parcellation from resting-state correlations,” Cereb. Cortex 26(1), 288–303 (2016). 53OPAV 1047-3211 10.1093/cercor/bhu239 25316338 PMC4677978

[40] Shimizu T. Karten H. J., “Immunohistochemical analysis of the visual wulst of the pigeon (*Columba livia*),” J. Comp. Neurol. 300(3), 346–369 (1990). JCNEAM 0021-9967 10.1002/cne.903000307 1979983

[r41] Long X. et al., “Functional connectivity‐based parcellation of the human sensorimotor cortex,” Eur. J. Neurosci. 39(8), 1332–1342 (2014). EJONEI 0953-816X 10.1111/ejn.12473 24417550

[r42] Behroozi M. et al., “Functional connectivity pattern of the internal hippocampal network in awake pigeons: a resting-state fMRI study,” Brain. Behav. Evol. 90(1), 62–72 (2017). BRBEBE 0006-8977 10.1159/000475591 28866684

[43] Schaefer A. et al., “Local-global parcellation of the human cerebral cortex from intrinsic functional connectivity MRI,” Cereb. Cortex 28(9), 3095–3114 (2018). 53OPAV 1047-3211 10.1093/cercor/bhx179 28981612 PMC6095216

[44] Eisen M. B. et al., “Cluster analysis and display of genome-wide expression patterns,” Proc. Natl. Acad. Sci. U. S. A. 95(25), 14863–14868 (1998). 10.1073/pnas.95.25.14863 9843981 PMC24541

[45] Behroozi M. et al., “In vivo measurement of T1 and T2 relaxation times in awake pigeon and rat brains at 7T,” Magn. Reson. Med. 79(2), 1090–1100 (2018). MRMEEN 0740-3194 10.1002/mrm.26722 28474481

[46] Jenkinson M. et al., “FSL,” NeuroImage 62(2), 782–790 (2012). NEIMEF 1053-8119 10.1016/j.neuroimage.2011.09.015 21979382

[r47] Smith S. M. et al., “Advances in functional and structural MR image analysis and implementation as FSL,” NeuroImage 23, S208–S219 (2004). NEIMEF 1053-8119 10.1016/j.neuroimage.2004.07.051 15501092

[48] Woolrich M. W. et al., “Bayesian analysis of neuroimaging data in FSL,” NeuroImage 45(1), S173–S186 (2009). NEIMEF 1053-8119 10.1016/j.neuroimage.2008.10.055 19059349

[49] Cox R. W., “AFNI: software for analysis and visualization of functional magnetic resonance neuroimages,” Comput. Biomed. Res. 29(3), 162–173 (1996). 10.1006/cbmr.1996.0014 8812068

[50] Cox R. W. Hyde J. S., “Software tools for analysis and visualization of fMRI data,” NMR Biomed. 10(4–5), 171–178 (1997). 10.1002/(SICI)1099-1492(199706/08)10:4/5<171::AID-NBM453>3.0.CO;2-L 9430344

[51] Avants B. Tustison N. J. Song G., “Advanced normalization tools: V1.0,” Insight J. (2009).

[52] Avants B. B. et al., “The optimal template effect in hippocampus studies of diseased populations,” NeuroImage 49(3), 2457–2466 (2010). NEIMEF 1053-8119 10.1016/j.neuroimage.2009.09.062 19818860 PMC2818274

[53] Tustison N. J. et al., “N4ITK: improved N3 bias correction,” IEEE Trans. Med. Imaging 29(6), 1310–1320 (2010). ITMID4 0278-0062 10.1109/TMI.2010.2046908 20378467 PMC3071855

[54] Galiakhmetova D. et al., “Ultra-short laser pulses propagation through mouse head tissues: experimental and computational study,” IEEE J. Sel. Top. Quantum Electron. 29(4: Biophotonics), 1–11 (2023). IJSQEN 1077-260X 10.1109/JSTQE.2022.3214788

[55] Libby L. A. et al., “Differential connectivity of perirhinal and parahippocampal cortices within human hippocampal subregions revealed by high-resolution functional imaging,” J. Neurosci. 32(19), 6550–6560 (2012). JNRSDS 0270-6474 10.1523/JNEUROSCI.3711-11.2012 22573677 PMC3374643

[56] Zhong Q. et al., “Functional parcellation of the hippocampus from resting-state dynamic functional connectivity,” Brain Res. 1715, 165–175 (2019). BRREAP 0006-8993 10.1016/j.brainres.2019.03.023 30910629

[57] Meunier D., “Hierarchical modularity in human brain functional networks,” Front. Neuroinf. 3, 37 (2009). 10.3389/neuro.11.037.2009 PMC2784301 19949480

[58] Atoji Y. Wild J. M., “Fiber connections of the hippocampal formation and septum and subdivisions of the hippocampal formation in the pigeon as revealed by tract tracing and kainic acid lesions,” J. Comp. Neurol. 475(3), 426–461 (2004). JCNEAM 0021-9967 10.1002/cne.20186 15221956

[r59] Atoji Y. Wild J. M., “Afferent and efferent connections of the dorsolateral corticoid area and a comparison with connections of the temporo-parieto-occipital area in the pigeon (Columba livia),” J. Comp. Neurol. 485(2), 165–182 (2005). JCNEAM 0021-9967 10.1002/cne.20490 15776448

[r60] Atoji Y. Wild J. M., “Anatomy of the avian hippocampal formation,” Rev. Neurosci. 17(1–2), 3–15 (2006). 10.1515/revneuro.2006.17.1-2.3 16703939

[r61] Herold C. et al., “The hippocampus of birds in a view of evolutionary connectomics,” Cortex 118, 165–187 (2019). 10.1016/j.cortex.2018.09.025 30442359

[62] Rook N. et al., “Neuronal circuits within the homing pigeon hippocampal formation,” J. Comp. Neurol. 531(7), 790–813 (2023). JCNEAM 0021-9967 10.1002/cne.25462 36808394

[63] Gordon E. M. et al., “Precision functional mapping of individual human brains,” Neuron 95(4), –791 (2017). NERNET 0896-6273 10.1016/j.neuron.2017.07.011 28757305 PMC5576360

[64] Fox M. D. et al., “The global signal and observed anticorrelated resting state brain networks,” J. Neurophysiol. 101(6), 3270–3283 (2009). JONEA4 0022-3077 10.1152/jn.90777.2008 19339462 PMC2694109

[65] Salvador R. et al., “Neurophysiological architecture of functional magnetic resonance images of human brain,” Cereb. Cortex 15(9), 1332–1342 (2005). 53OPAV 1047-3211 10.1093/cercor/bhi016 15635061

[66] Layden E. A. et al., “Interhemispheric functional connectivity in the zebra finch brain, absent the corpus callosum in normal ontogeny,” NeuroImage 195, 113–127 (2019). NEIMEF 1053-8119 10.1016/j.neuroimage.2019.03.064 30940612

[67] Power J. D. et al., “Spurious but systematic correlations in functional connectivity MRI networks arise from subject motion,” NeuroImage 59(3), 2142–2154 (2012). NEIMEF 1053-8119 10.1016/j.neuroimage.2011.10.018 22019881 PMC3254728

[68] Letzner S. Simon A. Güntürkün O., “Connectivity and neurochemistry of the commissura anterior of the pigeon (*Columba livia*),” J. Comp. Neurol. 524(2), 343–361 (2016). JCNEAM 0021-9967 10.1002/cne.23858 26179777 PMC5049482

[69] Schubert E., “Stop using the elbow criterion for k-means and how to choose the number of clusters instead,” ACM SIGKDD Explor. Newsl. 25(1), 36–42 (2023). 10.1145/3606274.3606278

[70] Cohen A. L. et al., “Defining functional areas in individual human brains using resting functional connectivity MRI,” NeuroImage 41(1), 45–57 (2008). NEIMEF 1053-8119 10.1016/j.neuroimage.2008.01.066 18367410 PMC2705206

[71] Williams A. H. et al., “Unsupervised discovery of demixed, low-dimensional neural dynamics across multiple timescales through tensor component analysis,” Neuron 98(6), 1099–1115.e8 (2018). NERNET 0896-6273 10.1016/j.neuron.2018.05.015 29887338 PMC6907734

[72] Grandjean J. et al., “Optimization of anesthesia protocol for resting-state fMRI in mice based on differential effects of anesthetics on functional connectivity patterns,” NeuroImage 102, 838–847 (2014). NEIMEF 1053-8119 10.1016/j.neuroimage.2014.08.043 25175535

[73] Van Ruijssevelt L. et al., “Auditory evoked BOLD responses in awake compared to lightly anaesthetized zebra finches,” Sci. Rep. 7(1), 13563 (2017). SRCEC3 2045-2322 10.1038/s41598-017-13014-x 29051552 PMC5648849

[74] Fishell A. K. et al., “Portable, field-based neuroimaging using high-density diffuse optical tomography,” NeuroImage 215, 116541 (2020). NEIMEF 1053-8119 10.1016/j.neuroimage.2020.116541 31987995

[75] Wheelock M. D. Culver J. P. Eggebrecht A. T., “High-density diffuse optical tomography for imaging human brain function,” Rev. Sci. Instrum. 90(5), 051101 (2019). RSINAK 0034-6748 10.1063/1.5086809 31153254 PMC6533110

[76] Stacho M. et al., “A cortex-like canonical circuit in the avian forebrain,” Science 369(6511), eabc5534 (2020). SCIEAS 0036-8075 10.1126/science.abc5534 32973004

[77] Eggebrecht A. T. et al., “Mapping distributed brain function and networks with diffuse optical tomography,” Nat. Photonics 8(6), 448–454 (2014). NPAHBY 1749-4885 10.1038/nphoton.2014.107 25083161 PMC4114252

[78] Zeff B. W. et al., “Retinotopic mapping of adult human visual cortex with high-density diffuse optical tomography,” Proc. Natl. Acad. Sci. U. S. A. 104(29), 12169–12174 (2007). 10.1073/pnas.0611266104 17616584 PMC1924577

[79] Hayashi R. et al., “Diffuse optical tomography using fNIRS signals measured from the skull surface of the Macaque monkey,” Cereb. Cortex Commun. 3(1), tgab064 (2022). 10.1093/texcom/tgab064 35072075 PMC8767783

[80] Culver J. P. et al., “Diffuse optical tomography of cerebral blood flow, oxygenation, and metabolism in rat during focal ischemia,” J. Cereb. Blood Flow Metab. 23(8), 911–924 (2003). 10.1097/01.WCB.0000076703.71231.BB 12902835

[81] Meek J. H. et al., “Regional haemodynamic changes in the occipital cortex of awake infants due to visual stimulation,” NeuroImage 7(4), S311 (1998). NEIMEF 1053-8119 10.1016/S1053-8119(18)31144-3

[r82] Taga G. et al., “Brain imaging in awake infants by near-infrared optical topography,” Proc. Natl. Acad. Sci. U. S. A. 100(19), 10722–10727 (2003). 10.1073/pnas.1932552100 12960368 PMC196871

[r83] Sakatani K. et al., “Cerebral blood oxygenation changes induced by auditory stimulation in newborn infants measured by near infrared spectroscopy,” Early Hum. Dev. 55(3), 229–236 (1999). EHDEDN 0378-3782 10.1016/S0378-3782(99)00019-5 10463787

[r84] Gibson A. P. et al., “Three-dimensional whole-head optical tomography of passive motor evoked responses in the neonate,” NeuroImage 30(2), 521–528 (2006). NEIMEF 1053-8119 10.1016/j.neuroimage.2005.08.059 16246586

[85] Debracque C. et al., “Validating the use of functional near-infrared spectroscopy in monkeys: the case of brain activation lateralization in Papio anubis,” Behav. Brain Res. 403, 113133 (2021). BBREDI 0166-4328 10.1016/j.bbr.2021.113133 33482169

[86] Brier L. M. Culver J. P., “An open source statistical and data processing toolbox for wide-field optical imaging in mice,” Neuroscience (2021). 10.1117/1.NPh.10.1.016601 PMC9976616 36874217

[87] Brier L. M. Culver J. P., “Open-source statistical and data processing tools for wide-field optical imaging data in mice,” Neurophotonics 10(01), 016601 (2023). 10.1117/1.NPh.10.1.016601 36874217 PMC9976616

