# Supplementary material for: Mapping functional connectivity in the pigeon brain with wide-field optical imaging
Source: Neurophotonics. 2026 Feb 6;13(1):015010. doi: 10.1117/1.NPh.13.1.015010 (PMC12879446; doi:10.1117/1.NPh.13.1.015010)
Supplement: Supplementary file 1 [file NPh_013_015010_SD001.pdf]

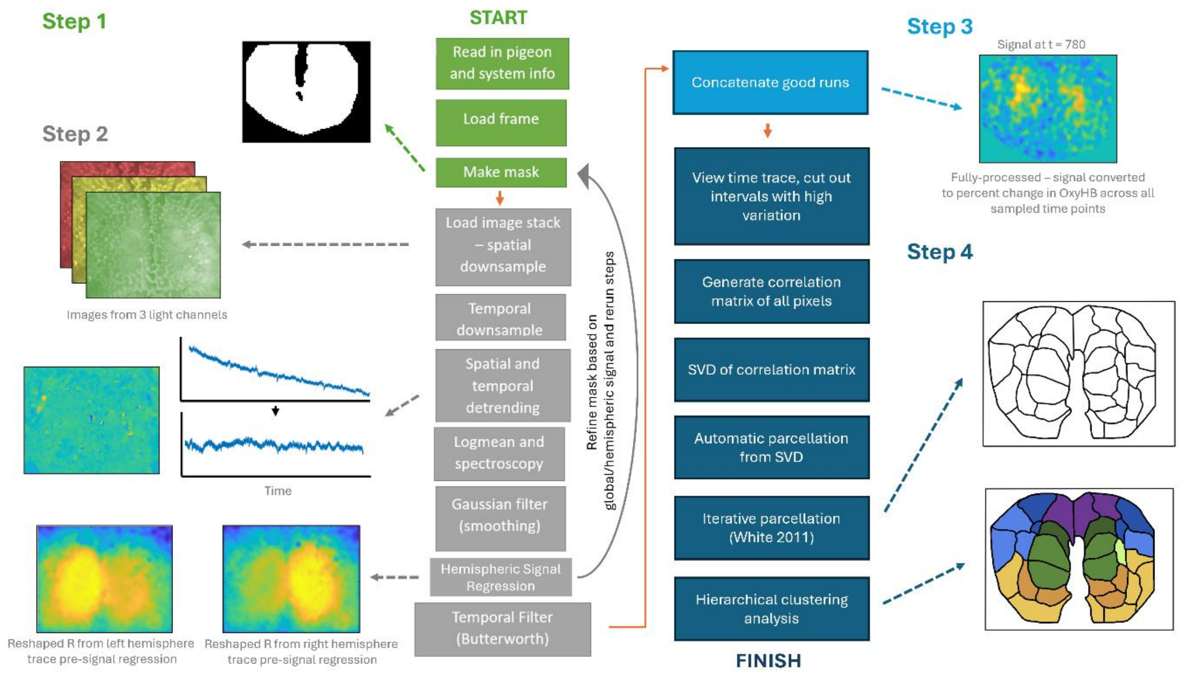

Fig. S1. Flowchart of steps in WOI processing pipeline. The initial processing steps (steps 1 and 2 at left) in this pipeline are adapted from the original used in Ref. 87, while steps 3 and 4 follow the iterative parcellation method used in Ref. 10 (see main text). Next to selected steps we show visual examples (highlighted with dotted arrows) of the evolution of the signal within our viewing window from raw data into final parcellation. Note that not all images used as examples in this pipeline are from the same brain.

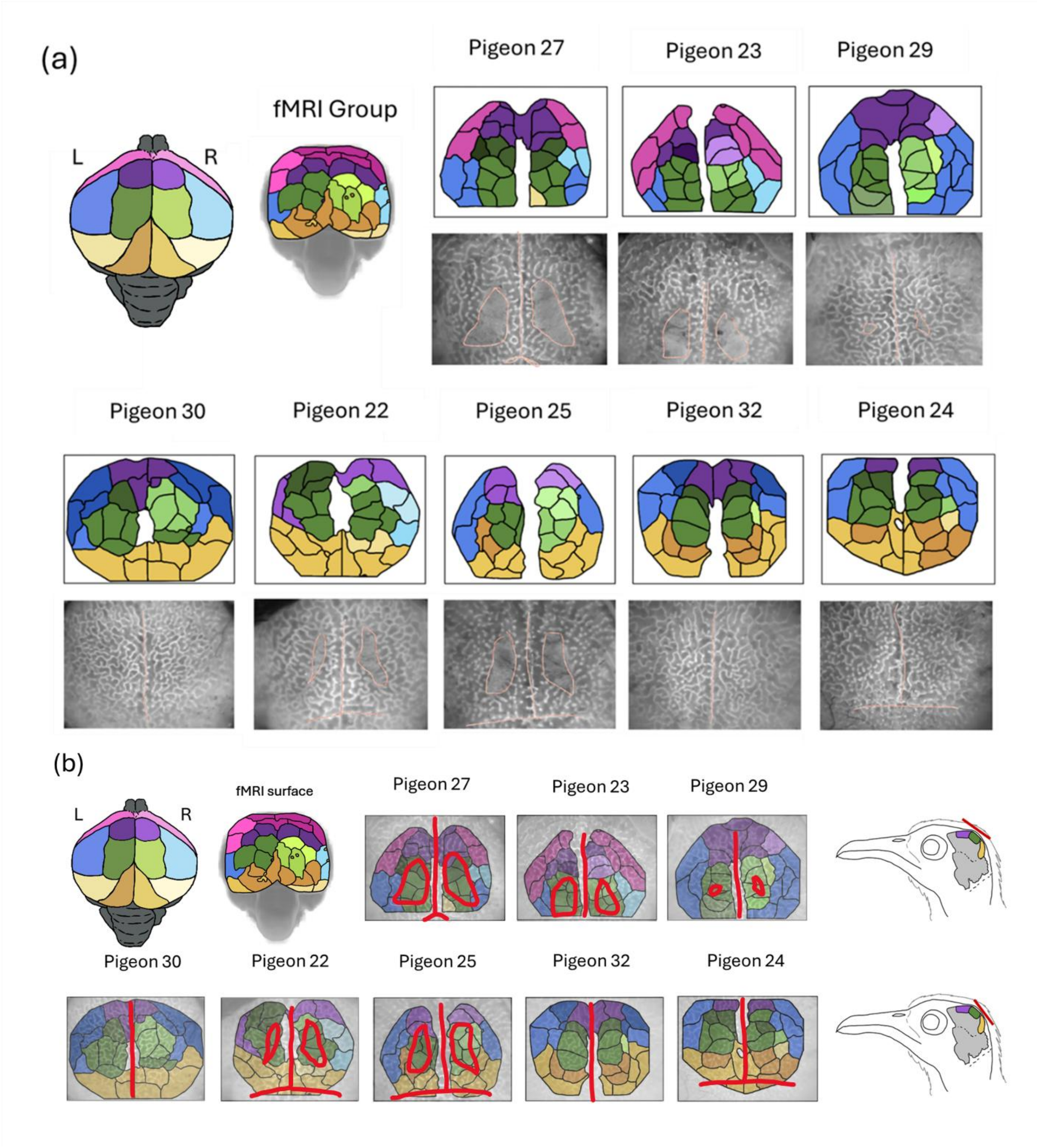

Fig. S2. White light images of exposed skull, a) next to and b) superimposed on WOI parcellation maps. In A, skull suture lines and the thinner/ transparent patch which occasionally appears over the visual Wulst are outlined in light pink (the visibility of these skull landmarks are often dependent on pigeon age). In B, the white light images are overlaid on the parcellation maps and made transparent, with the same skull features drawn in red for clarity.

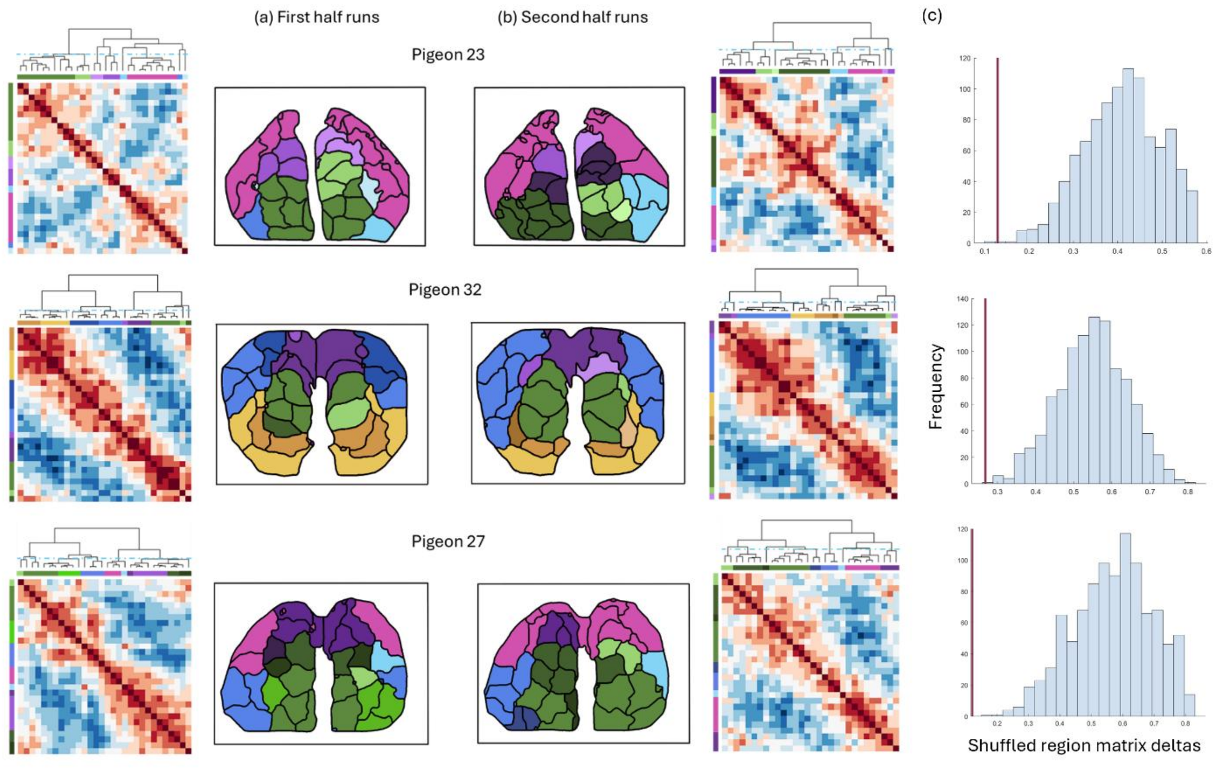

Fig. S3. Within-individual variation in high-level parcel associations. Breakdown of cluster associations from separate iterative parcellation and hierarchical clustering analysis comparing two five-minute runs: one run from (a) the first half and (b) one run from the second half of a WOI scanning session for three individuals. (c) Observed deltas between region-scale matrices from first and second half parcellations for each individual (maroon lines at left superimposed on) were significantly smaller than would be expected by chance given randomly-shuffled matrix delta distribution (probability of less than or equal to our observed value: Pigeon 32 and pigeon 27, $P < 0.001$; pigeon 23, $P = 0.001$).

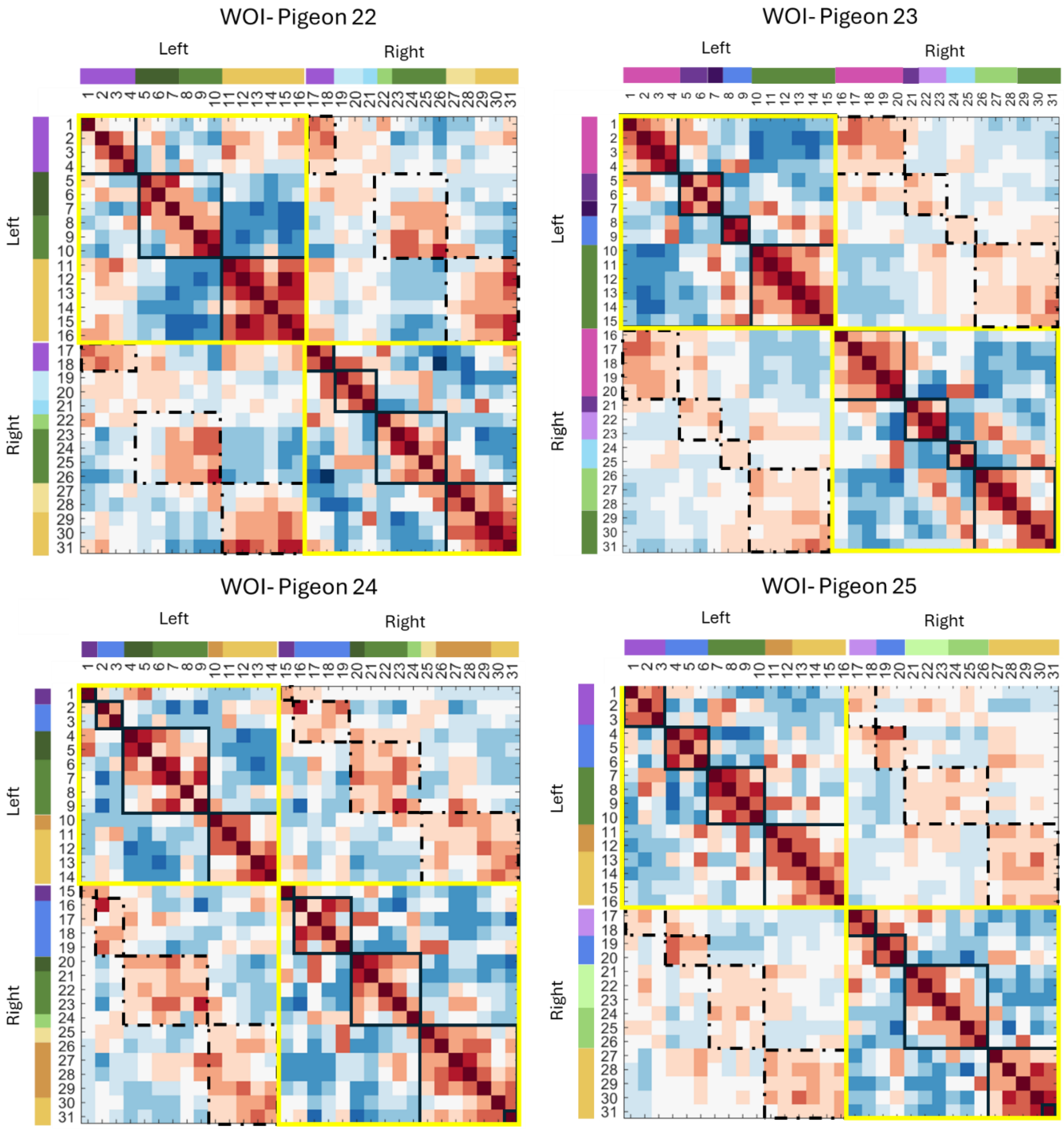
WOI- Pigeon 22
Left
Right
WOI- Pigeon 23
Left
Right
WOI- Pigeon 24
Left
Right
WOI- Pigeon 25
Left
Right

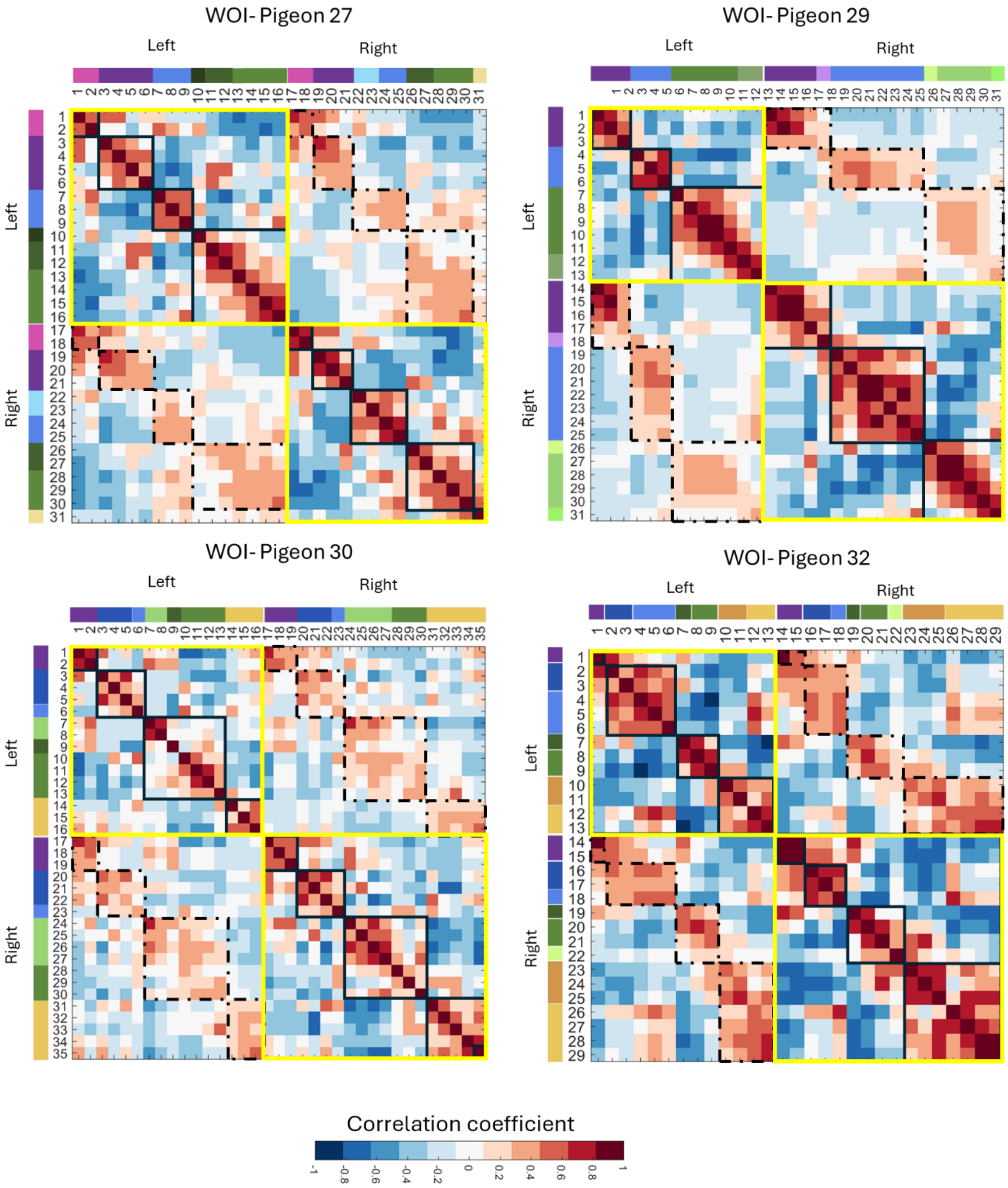

Fig. S4. Individual correlation matrices for all WOI individuals, with parcels split by hemisphere and reordered by cluster identity. Cluster color assignments for each parcel are shown in bars at left and top of matrix next to parcel numbers. Data from within a single hemisphere (top left and bottom right) are highlighted with yellow squares, whereas data from high-level clusters in the same anatomical region are highlighted with smaller black squares. Lower left and upper right quadrants in these matrices depict cross-hemisphere correlations; homotopic comparisons within them are highlighted with dashed rectangles. Relationships above the diagonal are identical to those below it. Overall, all clusters from all individuals exhibit positive homotopic correlations, showing robustness of these relationships across individuals.

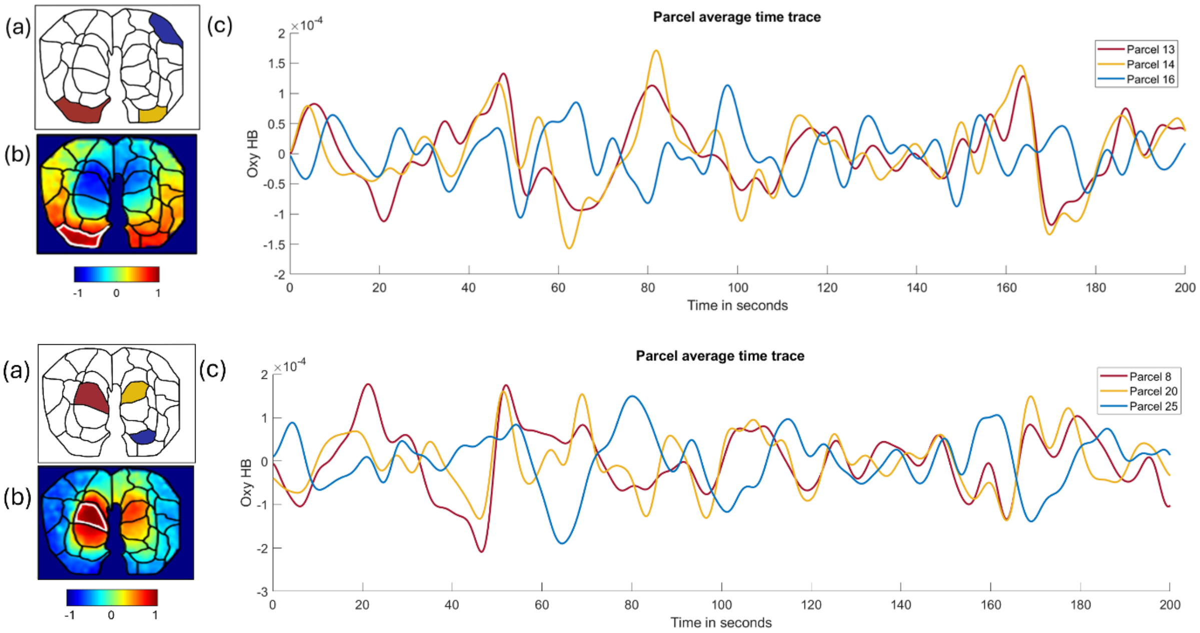

Fig. S5. Correlation patterns in average parcel activity from three selected regions in the pigeon brain. (a) Focal parcels are highlighted in red, yellow, and blue (red and yellow are homotopic, and form part of the hippocampus in the top example and the visual Wulst in the bottom example). (b) Functional connectivity maps for the red parcels in (a). Colors depict Pearson's correlation coefficient, with redder colored pixels depicting more similar activity to the parcel. (c) Average time traces (i.e., observed changes in oxygenated hemoglobin levels) for these three parcels. The red and yellow parcels are positively correlated with each other (r = 0.767 for the top hippocampus example and r = 0.626 for the bottom visual Wulst example) and uncorrelated with the blue parcel (e.g., red v. blue: Top example r = -0.077, Bottom r = -0.054).
